# Lower glycolysis carries a higher flux than any biochemically possible alternative

**DOI:** 10.1038/ncomms9427

**Published:** 2015-09-29

**Authors:** Steven J. Court, Bartlomiej Waclaw, Rosalind J. Allen

**Affiliations:** 1 SUPA, School of Physics and Astronomy, University of Edinburgh, James Clerk Maxwell Building, Peter Guthrie Tait Road, Edinburgh EH9 3FD, UK

## Abstract

The universality of many pathways of core metabolism suggests a strong role for evolutionary selection, but it remains unclear whether existing pathways have been selected from a large or small set of biochemical possibilities. To address this question, we construct *in silico* all possible biochemically feasible alternatives to the trunk pathway of glycolysis and gluconeogenesis, one of the most highly conserved pathways in metabolism. We show that, even though a large number of alternative pathways exist, the alternatives carry lower flux than the real pathway under typical physiological conditions. We also find that if physiological conditions were different, different pathways could outperform those found in nature. Together, our results demonstrate how thermodynamic and biophysical constraints restrict the biochemical alternatives that are open to evolution, and suggest that the existing trunk pathway of glycolysis and gluconeogenesis may represent a maximal flux solution.

## Abstract (web summary)

The biochemical pathways of central carbon metabolism are highly conserved across all domains of life. Here, Court *et al*. use a computational approach to test all possible pathways of glycolysis and gluconeogenesis and find that the existing trunk pathways may represent a maximal flux solution selected for during evolution.

## Graphical abstract

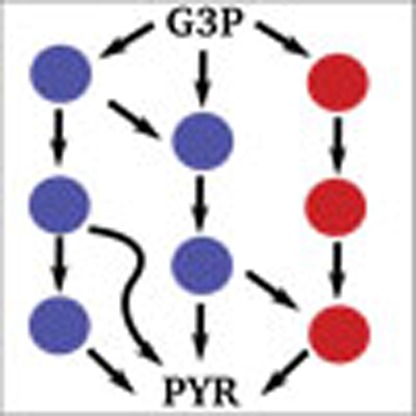

The biochemical pathways of central carbon metabolism are highly conserved across all domains of life, and largely control the productivity of life on the Earth^1^,^2^. Yet it remains unknown whether these pathways are the result of historical contingency during early evolution^3^,^4^, or are instead optimal solutions to the problem of energy and biomass production^5^,^6^,^7^,^8^,^9^,^10^. Put simply, are there alternative biochemically feasible pathways that could perform the same function, and if so, how do they perform compared with those found in nature?

Previous studies have mainly addressed this question either by constructing simplified artificial metabolic networks^5^,^6^,^7^, or by mining databases of biochemical compounds and reactions for known organisms^4^,^8^,^9^,^11^,^12^,^13^. Both of these approaches have drawbacks: the former does not capture real biochemistry, whereas the latter is limited to metabolites and reactions found in well-studied organisms. Nevertheless, this work has led to suggested optimality principles including maximizing biochemical flux or yield^6^,^7^, minimizing biochemical steps or protein costs^5^,^8^ and ensuring that function is maintained in a changing environment^11^. Only a few studies have attempted to explore the full universe of possible metabolic pathways, using realistic rules of biochemistry^10^,^14^,^15^,^16^,^17^. In particular, using this approach, Noor *et al*.^10^ recently suggested that central carbon metabolism, taken as a whole, uses the minimal number of enzymatic steps needed to generate a predefined set of biochemical precursors.

Here, we perform an exhaustive computational search for all possible biochemically feasible alternatives to one of the most ancient and most highly conserved sections of central carbon metabolism, the trunk pathway of glycolysis and gluconeogenesis. Glycolysis breaks down glucose to pyruvate, generating ATP, NADH and biosynthetic precursors, whereas gluconeogenesis uses ATP and NADH to generate glucose from pyruvate. The glycolytic-gluconeogenic pathway is almost linear and can be divided into two parts: an ‘upper' chain of reactions involving 6-carbon molecules, which connects glucose to glyceraldehyde-3-phosphate (G3P), and a ‘lower' chain of reactions involving 3-carbon molecules, which connects G3P to pyruvate (Fig. 1). This lower reaction chain is known as the trunk pathway. In prokaryotes, the glycolytic and gluconeogenic trunk pathways consist of almost the same set of five reactions, differing only in one step. In glycolysis, the exergonic conversion of phosphoenolpyruvate (PEP) to pyruvate is coupled to the phosphorylation of ADP to ATP, whereas in gluconeogenesis the reverse reaction is driven by the hydrolysis of ATP to AMP, either with release of inorganic phosphate (the phosphoenolpyruvate synthase (pps) route) or via consumption of inorganic phosphate and release of pyrophosphate (the phosphate dikinase (ppdk) route).

Although the upper part of the glycolytic/gluconeogenic pathway exists in several very distinct variants, most notably the Embden–Meyerhof–Parnas (EMP) and Entner–Doudoroff (ED) pathways (Supplementary Fig. 1)^8^,^18^, the trunk pathway is near-ubiquitous, and contains enzymes which are highly conserved and universally distributed across the three domains of life^19^,^20^. Only a very small number of variations are observed in nature (see Supplementary Discussion for details). The existence of such an ancient and universal pathway suggests three possible scenarios: (i) the trunk pathway is the only biochemical possibility, (ii) alternatives exist but the extant pathway is evolutionarily optimal and (iii) alternatives are possible but have not been found by evolution. Distinguishing these scenarios lies at the heart of much of evolutionary biology. Here, we address this question directly using a computational approach. By systematically constructing and exploring the full space of biochemically feasible metabolites and reactions, many of which are not currently exploited by any known organism, we find that hundreds of alternative trunk pathways are possible. The one observed in nature, however, carries the maximal biochemical flux, under reasonable constraints on the intermediate metabolite and enzyme concentrations. Our results suggest that the trunk pathway represents an optimal solution among many possible alternatives.

## Results

### A network of all possible biochemical reactions

We used a computer program to generate the network of all possible biochemically feasible pathways between G3P and pyruvate.

#### Metabolites

Our program first systematically generates all possible relevant internal metabolites, that is, molecules intermediate between G3P and pyruvate, including those that are not found in nature (see Methods for details). Central carbon metabolism consists exclusively of reactions between ‘CHOPN' molecules: those composed of carbon, hydrogen, oxygen, phosphorus and nitrogen atoms, with phosphorus being present only in phosphate groups^21^,^22^. Moreover, the trunk pathway contains only unbranched aliphatic 3-carbon CHOP molecules. For completeness, we therefore include in our analysis all possible unbranched aliphatic CHOPN molecules containing up to four carbon atoms. We consider only molecules that are negatively charged (that is, include carboxyl or phosphate groups). This condition is motivated by the need to avoid leakage through the negatively charged phospho-lipid membrane, and is satisfied by almost all molecules in core metabolism^23^. Applying these criteria results in almost 1,500 different molecules, including all the internal metabolites in the real trunk pathway. We computed the free energy of formation Δ_*f*_*G* for all our internal metabolites, using existing experimental data where possible^24^ or, in the absence of such data, using a variant of the group contribution method^25^,^26^,^27^,^28^ (see Methods for details).

#### Reactions

Our program generates all possible reactions among our set of internal metabolites, based on the 20 Enzyme Commission (EC) reaction classes^29^ which encompass all the reactions between CHOPN molecules in core metabolism, of length four carbon atoms or less (Table 1). Many of these reactions involve cofactors such as ATP and NAD^+^; we refer to these as external metabolites and we assume that they have fixed concentrations, which define the cellular (physiological) conditions. The full set of external metabolites in our reaction network is shown in Supplementary Table 1 and includes the energy currency metabolite ATP, the redox cofactor NAD^+^, the amino-group donor glutamate, as well as inorganic phosphates and CO_2_.

### Our network generates many alternative trunk pathways

Our network reveals a huge number of alternative pathways connecting G3P and pyruvate, which are consistent with the rules of biochemistry. Enumerating these alternatives, we find 2, 29, 555 and 5,859 pathways of length 3, 4, 5 and 6, respectively, with the number of pathways increasing exponentially with path length (Supplementary Fig. 2). Some of these alternative pathways are found to use the same set of reaction types as the natural trunk pathway, but execute them in a different order (for example, left pathway of Fig. 2a), whereas others make use of a different set of reaction types (right pathway of Fig. 2a).

We wish to investigate how many of these alternative trunk pathways are feasible under typical physiological conditions. First, in the glycolytic direction, we demand that candidate pathways produce at least two ATP molecules. This is required so that the full glycolytic pathway produces a net ATP yield, as the two dominant forms of glycolysis, the EMP and ED pathways, consume one ATP molecule per G3P in their upper halves. In addition, motivated by considerations of simplicity^5^,^10^ and cost of enzyme production^8^, we further restrict our analysis to those pathways with a near-minimal number of steps. This results in a set of 1,787 candidate glycolytic pathways of length 4, 5 and 6 that produce at least two ATP molecules, and a set of 6,445 biochemically plausible gluconeogenic pathways of lengths 3–6 (not taking into account thermodynamics).

### The real paths maximize flux under physiological conditions

**Figure 1:**
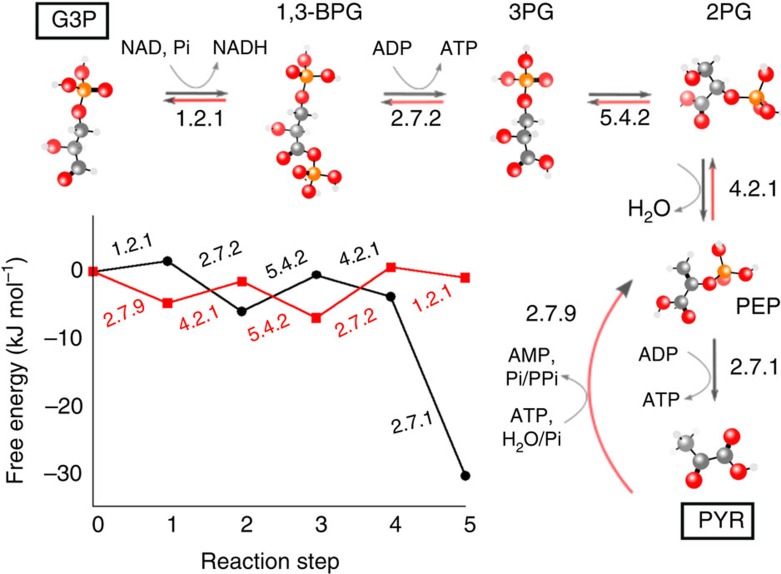
The trunk pathway of glycolysis and gluconeogenesis. The end points are glyceraldehyde 3-phosphate (G3P) and pyruvate (PYR); intermediate metabolites are 1,3-bisphosphoglycerate (1,3-BPG); 3-phosphoglycerate (3-PG); 2-phosphoglycerate (2-PG) and phosphoenolpyruvate (PEP). For each reaction, the external metabolites involved and the first three numbers from the EC number classification are indicated. The inset shows thermodynamic profiles for the trunk pathway in the glycolytic and gluconeogenenic (pps) directions (see Methods for details).

We next evaluated the performance of the alternative trunk pathways generated by our network, by comparing their steady-state metabolic flux. For glycolytic pathways, the metabolic flux corresponds to the rate of ATP production, whereas for gluconeogenic pathways, it corresponds to the rate of production of G3P (and ultimately of glucose). This flux depends not only on the total free energy change across a given pathway, but also on the distribution of the individual reaction free energies (see, for example, Fig. 1), which in turn depends on the intracellular environment via the concentrations of the external metabolites. For linear pathways, the flux can be calculated analytically, assuming linear kinetics with diffusion-limited enzymes^7^,^30^,^31^ (see Methods for details). We also assume that, for each pathway, the individual enzyme concentrations are optimized to maximize pathway flux, for a fixed total amount of enzyme (see Methods for details).

Importantly, when calculating the metabolic flux, we impose constraints on the intermediate internal metabolite concentrations. For the bacterium *Escherichia coli*, metabolite concentrations range from 0.1 μM to 100 mM, with the total intracellular metabolite pool being around 300 mM (ref. 32). We expect that very low metabolite concentrations are undesirable because of vanishing fluxes, whereas very high concentrations are precluded by osmotic considerations. In our initial comparison, we therefore set the flux to zero for a given pathway if any of its intermediate metabolite concentrations falls outside the range of 0.1 μM–100 mM.

Imposing this constraint, we calculated the metabolic flux of all our candidate pathways, across a wide range of intracellular conditions, as defined by the external metabolite concentrations. To explore the performance of our candidate pathways over a wide range of possible intracellular conditions, we randomly selected 10,000 points from the parameter space consisting of the concentrations of the 11 external metabolites, G3P and pyruvate, sampling each parameter logarithmically over several orders of magnitude above and below its typical physiological concentration (see Methods for details). For each point in parameter space, we evaluated the flux of each candidate pathway.

**Figure 2:**
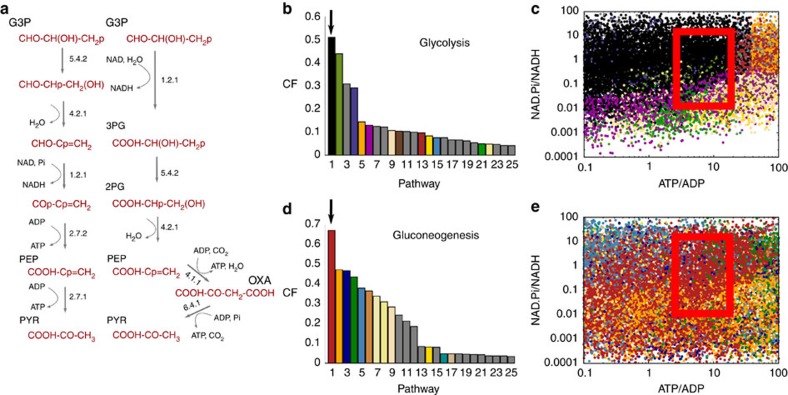
The real glycolytic and gluconeogenic trunk pathways represent maximal flux solutions. (**a**) Two alternative glycolytic pathways; left-hand pathway uses the same set of reaction classes, defined by EC number, as the real trunk pathway, whereas the right-hand pathway uses a different set. (**b**–**d**) Results of pathway comparison. (**b**,**d**) Alternative pathways generated in our analysis ordered by their comparative flux (see text for details), averaged across the whole parameter space. The top 25 paths are shown. (**b**) Glycolytic pathways; the black bar (indicated by the arrow) is the natural pathway. (**d**) Gluconeogenic paths; the red bar (indicated by the arrow) is the natural pps route. (**c**,**e**) Relative pathway performance as a function of the intracellular environment. Each dot represents a randomly sampled point in parameter space; the colour of the dot indicates the candidate pathway, which had the highest flux at that point in parameter space with colours corresponding to **b** and **d**. For parameter sampling procedure, see Methods. The axes correspond to two important characteristic measures of a cell, the redox state and energy state, via [NAD][Pi]/[NADH] and [ATP]/[ADP], respectively. The red boxes indicate the typical physiological state of the cell^32^,^43^: [ATP]/[ADP]=3–20, [NAD][Pi]/[NADH]=0.01–10 M (based on [NAD]/[NADH]=10–100 and [Pi]=1–100 mM). (**c**) Natural glycolytic pathway (black) tends to perform best under typical physiological conditions. (**e**) Natural pps gluconeogenic pathway (red) tends to perform best under typical cellular conditions. See Supplementary Data 1 and 2 for pathway structures.

As a simple metric, we first compare the performance of our candidate pathways, averaged over the entire parameter space. To this end, we compute the comparative flux (CF), which is the flux of a given pathway, divided by the maximum flux obtained among all pathways, at a given point in parameter space. Averaging this quantity across the whole parameter space gives a measure of relative performance for each candidate path. Based on this metric, we find that the real glycolytic and gluconeogenic pathways perform remarkably well, compared with the many alternatives (Fig. 2b,d). For glycolysis (Fig. 2b and Supplementary Data 1), the natural trunk pathway (in black, indicated by the arrow) outperforms all the alternative pathways; whereas for gluconeogenesis (Fig. 2d, Supplementary Data 2), one of the real pathways (the pps variant) is also ranked first (in red, indicated by the arrow). These results strongly suggest that the natural trunk pathways carry a high flux compared with alternatives; however, this metric is dependent on the range of parameter space that is sampled.

To overcome this issue and to investigate in more detail, we analysed which of our candidate pathways achieved the highest flux at different points in the parameter space. This allows us to understand how the performance of a given pathway depends on the intracellular environment. Our results show that different candidate pathways perform best in different regions of the parameter space. In particular, pathway performance is very sensitive to the cellular energy state, as measured by the ratio of the ATP and ADP concentrations ([ATP]/[ADP]), and redox state, represented by the ratio [NAD][Pi]/[NADH] (Fig. 2c,e). Focusing on the glycolytic pathways (Fig. 2b) we see that, remarkably, the natural trunk pathway (black dots) outperforms all the alternatives in the region of parameter space close to that found in living cells (red box). This suggests that the glycolytic trunk pathway represents a maximal flux solution for the conversion of G3P to pyruvate, under typical intracellular conditions. Similarly, for the gluconeogenic pathways (Fig. 2d), the pps pathway found in nature (red dots) outperforms the alternatives under typical physiological conditions (red box).

### Alternative flux calculations

The flux calculation used above makes the simplifying assumption that all enzymes are perfect catalysts^6^,^7^,^30^,^31^ and thus it neglects the important physical feature of substrate saturation. Any attempt at applying a generic choice of kinetic equations to a system of reactions is problematic, and more complicated forms give rise to a greater number of parameters that must be specified. However, one can test the effect that using alternative forms has on the overall outcome, and we find that the above results still hold when two forms of reversible Michaelis–Menten kinetics are assumed (Supplementary Figs 3, 4 and Methods), capturing the behaviour of substrate and product inhibition. Thus, our results are not dependent on the detailed assumptions made in modelling the enzyme kinetics.

### Constraints on metabolite concentrations are important

Imposing constraints on the intermediate internal metabolite concentrations is crucial to our analysis. When these constraints are neglected, a very different set of pathways dominate over the parameter space, many of which contain individual reactions with large Δ_*r*_*G* values, and the real glycolytic pathway falls to number 87 with respect to its CF averaged over parameter space (Supplementary Note 1 and Supplementary Data 3). It is possible, however, that our limits on the concentrations are too severe, as in reality the internal structure of a cell, as well as phenomena such as substrate channelling, can give rise to effective concentrations much larger or smaller than any global limit. Nevertheless, Supplementary Fig. 5 along with Supplementary Data 4 and 5 demonstrates that our results still hold when we repeat our analysis with a more generous allowed concentration range of 1 nM–500 mM.

### Other paths can perform better under different conditions

**Figure 3:**
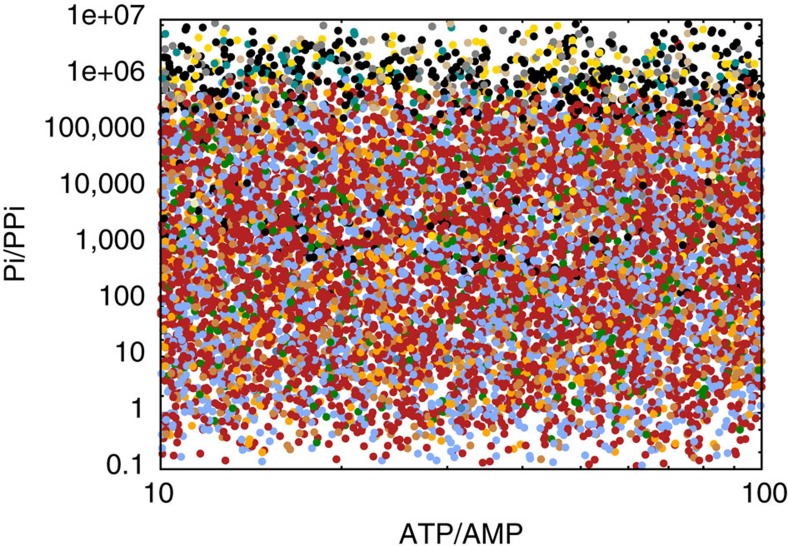
The sampled parameter ranges are an important factor in our analaysis. Repeating the comparison of candidate gluconeogenic paths, but now sampling [PPi] from 10^−8^ to 10^−2^ M instead of from 10^−4^ to 10^−2^ shows that the real ppdk pathway (black) becomes favourable compared with the pps pathway (red), when the ratio of orthophosphate to pyrophosphate concentrations is increased. Colours as in Fig. 2.

We expect the concentrations of the external metabolites to vary across organisms and across different environmental habitats. Hence, an interesting question to ask is could any alternative pathways outperform the real trunk pathways, if the physiological conditions were different? Indeed, we find that for some values of the external metabolite concentrations, other pathways outperform the natural ones (Fig. 2). Of particular interest is the fact that the pyrophosphate concentration is an important factor in our comparison of the gluconeogenic pathways. Figure 3 shows that at low PPi concentrations, the natural ppdk variant of the gluconeogenic pathway becomes more thermodynamically favourable and can outperform the more common pps pathway under typical physiological conditions. In nature, cellular pyrophosphate concentrations can be kept at sub-*μ*M levels through the highly efficient inorganic diphosphatase enzyme, EC 3.6.1.1.

### Alternative trunk pathways

**Figure 4:**
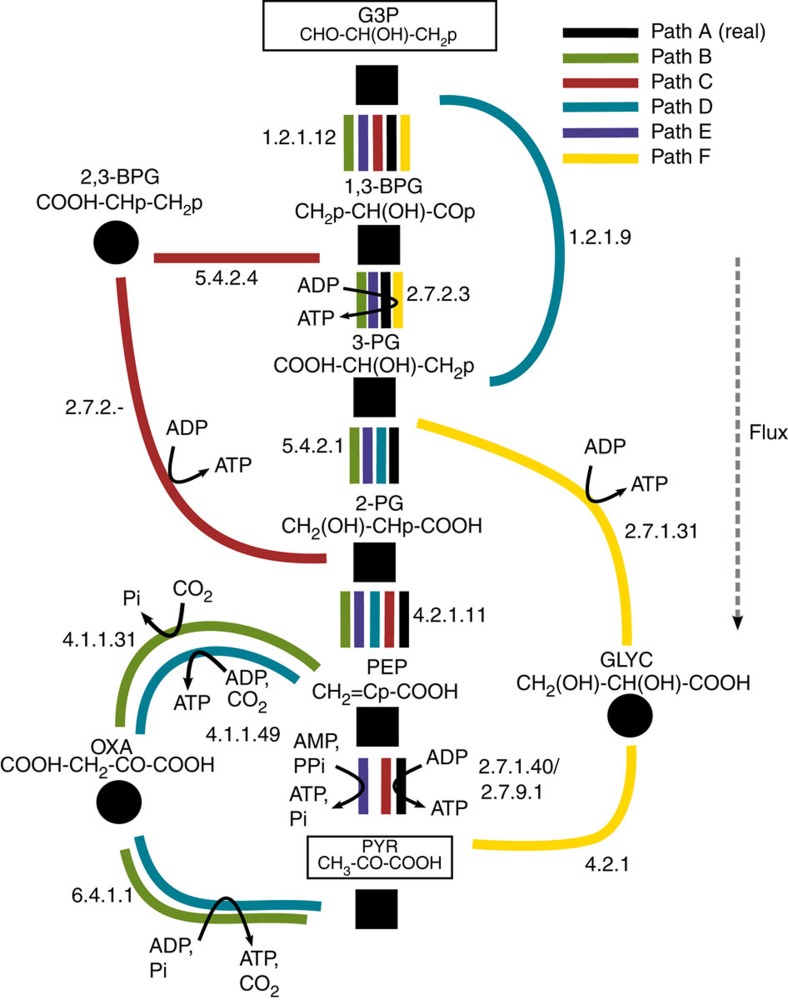
Thermodynamically feasible alternative glycolytic pathways utilizing compounds found in KEGG^35^ (see Supplementary Note 2 for more details). Reactions are indicated by coloured lines; full EC numbers in black correspond to enzymes present in KEGG, incomplete numbers are not found in KEGG. All of the enzymes for alternative Paths B, D and E are known to exist in nature. GLY, glycerate; OXA, oxaloacetate; 2,3-BPG=2,3-bisphosphoglycerate; all others as in Fig. 1.

It is interesting to investigate the alternative pathways revealed by our analysis in more detail. Our understanding of even the most well-studied metabolic pathways is incomplete, with new features being discovered in glycolysis^33^ and the pentose phosphate pathway^34^ in recent years. Hence, it is interesting to consider the alternatives found that most closely resemble the natural pathway, as it is possible that some of these exist in nature, but may be as yet uncharacterized. Figure 4 shows five thermodynamically feasible alternative glycolytic pathways found in our network, which utilize only chemical compounds found in the KEGG COMPOUND database of known metabolites^35^ (Supplementary Fig. 6 and Supplementary Data 6). In fact, in three of these pathways (B, D and E), the relevant enzyme for each reaction is known to exist in nature (Supplementary Note 2).

Path B is a pathway of length 6 and corresponds to the second-highest performing pathway (green) in Fig. 2b. This pathway follows the first four steps of the natural pathway before diverging at PEP to pass through oxaloacetate. This shunt first involves the carboxylation and dephosphorylation of PEP via PEP carboxylase EC 4.1.1.31, followed by a coupled decarboxylation and ATP-generation via pyruvate carboxylase EC 6.4.1.1.

Path C is similar to the real glycolytic trunk path, except that 1,3-bisphosphoglycerate (1,3-BPG) is first isomerized (to 2,3-BPG via EC 5.4.2.4) and then dephosphorylated (to 2-phosphoglycerate (2-PG), with ATP generation), rather than being first dephosphorylated and then isomerized as in the natural pathway. Interestingly, a similar pathway exists in red blood cells, where 2,3-BPG is produced from 1,3-BPG via the Rapoport–Leubering shunt^33^. In red blood cells, however, the 2,3-BPG is hydrolysed to either 3-phosphoglycerate (3-PG) or 2-PG without ATP generation^33^, thus sacrificing one ATP compared with the usual glycolytic pathway. Although no enzyme has been characterized that generates ATP from 2,3-BPG, this reaction does actually appear in the KEGG database, as R02664.

Path D differs from the natural pathway in that it converts G3P directly to 3-PG without the production of ATP, via EC 1.2.1.9. This large exorgonic initial step gives path D a favourable thermodynamic profile and results in the two ATP-generating steps being the final two reactions, catalysed by PEP carboxykinase EC 4.1.1.49 and pyruvate carboxylase EC 6.4.1.1. These two enzymes are usually associated with a gluconeogenic role in animals and their feasibility in the glycolytic direction is sensitive to the intracellular pH; it becomes increasingly thermodynamically favourable to produce two ATP molecules from PEP with decreasing pH (see Supplementary Note 2 for details).

Path E is identical to Path A with the exception that the final step generates ATP from AMP and pyrophosphate (via pyruvate phosphate dikinase, EC 2.7.9.1) rather than from ADP and Pi—that is, it is the exact reverse of the natural ppdk gluconeogenic pathway. This may result in an effectively greater energetic yield than the natural glycolytic pathway, as an ATP is recovered from an AMP directly, rather than through the action of the adenylate kinase enzyme (EC 2.7.4.3), which involves the consumption of another ATP molecule. This pathway actually corresponds to a glycolytic variant observed in some anaerobic eukaryotes, in which glycolysis is the primary mode of ATP production^36^. Its flux and feasibility will depend on the cellular [AMP] and [PPi] concentrations, which is not the case for the natural glycolytic pathway (which does not involve AMP or PPi).

Finally, Path F represents a pathway of length 4 that produces two ATP molecules. It converts 3-PG to glycerate with the production of ATP, via glycerate 3-kinase, EC 2.7.1.31. Although the final reaction of this pathway is not present in the KEGG database, this putative reaction is chemically rather simple—involving the dehydration of glycerate to enolpyruvate followed by a spontaneous tautomerization to pyruvate—and so it seems plausible that it might be catalysed by the uncharacterized, non-primary action of an existing enzyme.

## Discussion

Despite the huge variety and complexity of life on Earth, the biochemistry of core metabolism is remarkably universal. Our analysis shows that this universality does not arise from an absence of other possibilities. Using a systematic approach, we have identified many alternatives to perhaps the most highly conserved set of metabolic reactions, the glycolytic and gluconeogenic trunk pathways. Our alternative pathways obey the rules of biochemistry, carry positive flux under reasonable intracellular conditions, and satisfy reasonable constraints on metabolite concentrations. Remarkably, of all these alternatives, we find that the trunk pathway observed in nature carries the highest biochemical flux in both the glycolytic and gluconeogenic directions, for parameters that represent typical intracellular physiological conditions. Of the two variants of the prokaryotic gluconeogenic pathway that are found in nature, the pps route is the best performer across a wide parameter range, whereas the ppdk route is more sensitive to environmental conditions, requiring a low concentration of pyrophosphate (Fig. 3). The fact that our analysis identifies the natural pathways using only flux maximization combined with constraints on intermediate metabolite concentrations suggests that these factors are likely to have been important driving forces in the evolution of metabolism. Importantly, our key results are also independent of the detailed assumptions made in modelling the enzyme kinetics.

Flux maximization is widely recognized as an important concept in the study of metabolism; both from the perspective of glycolysis and more broadly^37^. Previous works addressing this issue for glycolysis^5^,^6^,^7^ have arrived at similar conclusions but have remained abstract in nature. For the first time, we attempt to expand on these fundamental and influential studies through the use of a realistic set of generic enzymatic rules along with the inclusion of thermodynamics. Our results add support to the picture that has been developing, and also suggest that evolutionary pressures on metabolic fluxes have to operate within the context of reasonable constraints on metabolite concentrations, and that neglecting these constraints can produce dramatically different outcomes. Our results also expand on the recent suggestion of Noor *et al*.^10^ that central carbon metabolism can be understood as a minimal walk between the set of metabolites essential for growth. We find that the requirement to produce a set of essential biochemical precursors is not sufficient to explain the biochemical structure of the natural trunk pathway, as alternative pathways are possible which produce the essential precursors with the same number of steps (for example, Path D, Fig. 4). Also, many of our alternative pathways produce very similar, but not identical, intermediates to those of the real trunk pathway and it is conceivable that these could be used as alternative precursors. Our results show that flux maximization provides a criterion by which these alternative minimal-length pathways may be distinguished. We also note that this criterion is not affected by ATP yield because alternative pathways producing three or more ATP molecules are never thermodynamically feasible under typical physiological conditions (Supplementary Fig. 7), hence there cannot be any trade-off between the yield and the flux in our model.

Our analysis also reveals alternative trunk pathways that can perform better than the natural one under different physiological conditions. Although some of these alternatives involve compounds and reactions that are not found in biochemical databases, others use enzymes that are known to exist in nature. The latter pathways (Fig. 4) are clearly plausible biochemically and thermodynamically. It could be that some of these pathways do operate glycolytically under some circumstances, and may be revealed by future biochemical investigations (see Supplementary Note 2 for details).

In this study, we have limited our analysis to pathways that start and end at G3P and pyruvate. Relaxing this requirement would certainly lead to many more alternative pathways for the generation of energy and for biosynthesis. Although it is also important to consider other factors, including the need for integration within a wider metabolic network, our analysis suggests that key principles underlying the structure of core metabolism may emerge from simple biochemical, thermodynamic and biophysical considerations.

Finally, we briefly discuss broader evolutionary implications of our work. Assuming that the trunk pathway can be replaced by any of our alternative pathways without affecting the rest of the metabolic network too much, the ATP flux could be used as a proxy for fitness, that is, the ability of the organism to outcompete other, similar to organisms. Endowed with a mutational graph of possible mutations between different pathways (different genotypes), this would create a fitness landscape in which the highest peaks correspond to local maxima of the ATP flux. An interesting question is whether the global maximum (the real pathway) is accessible, that is, can it be reached from anywhere in the landscape? Such fundamental evolutionary questions have been addressed recently using the full set of known biochemical reactions^4^. It would be interesting to repeat this analysis using our data, and to compare the fitness landscape obtained for our model to the landscapes of other evolutionary models^38^,^39^,^40^,^41^. This could show to what extent different computer models of artificial life are similar to one another, and perhaps even shed light on the structure of real fitness landscapes.

## Methods

### Chemical compounds and reactions

We created a list of chemical compounds with 2, 3 or 4 carbon atoms by generating all possible linear combinations of the 20 ‘building blocks' shown in Supplementary Table 2. Each of the building blocks was composed of a single carbon atom with associated oxygen, hydroxyl, hydrogen, phoshate and/or amino groups. Building blocks were connected together in linear chains by single or double bonds. This procedure created 1,966 linear molecules, 1,477 of which are electrostatically charged in solution, that is, containing at least one carboxyl or phosphate group. These 1,477 molecules are our internal metabolites. Next, for every possible pair of molecules from this list we checked systematically whether the reactions from Table 1 (see Supplementary Table 3 also for details) could transform one molecule into another, allowing for all possible couplings with the external metabolites. In this way, a network of 7,940 reactions was generated.

### Free energies of compounds and reactions

For those internal metabolites that are known biochemical species, standard free energies of formation Δ_*f*_*G* were taken from the literature^24^. For other internal metabolites, for which such data does not exist, we employed a variant of the group contribution method^25^,^26^,^27^,^28^ which accounts for the fact that molecules exist in solution as an equilibrium mixture of different ionic species. For each such molecule *g*_1_*g*_2_…*g*_*n*_, composed from building blocks {*g*_*i*_}, we calculated Δ_*f*_*G* using


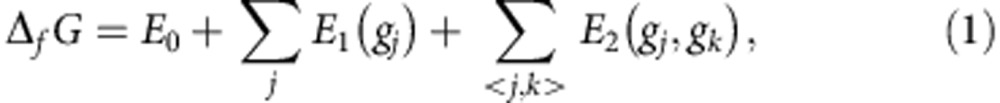


where *E*_0_ is a constant, *E*_1_(*g*_*j*_) is the contribution of group *g*_*j*_ and *E*_2_(*g*_*j*_,*g*_*k*_) is a small correction due to neighbouring group–group interactions. The values of *E*_0_, the vector *E*_1_ and matrix *E*_2_ are determined by performing a least-squares fit to a training set of molecules with known Δ_*f*_*G*s that correspond most closely to the linear CHOPN molecules of our network (see Supplementary Methods for details).

### Flux calculation

We used the method mentioned in ref. 7 to calculate the flux carried by a linear pathway. This method assumes that the flux through reaction *i* is given by^7^,^30^


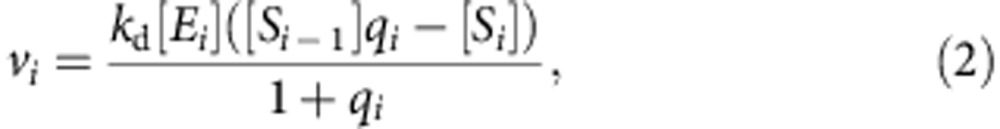


where *k*_d_ is the diffusion-controlled rate constant, [*E*_*i*_] is the enzyme concentration, [*S*_*i*−1_] and [*S*_*i*_] represent substrate and product concentrations and *q*_*i*_ is the thermodynamic constant. This expression assumes that the enzyme acts as a perfect catalyst, and is used to derive an expression for pathway flux and metabolite concentrations (see Supplementary Methods for details). We then use Powell's method^42^ to find the set of enzyme concentrations that maximize the flux subject to the constraints that (i) all steady-state intermediate concentrations are within the prescribed range and (ii) the total enzyme concentration is fixed. We also repeat our analysis using reversible Michaelis–Menten kinetics, see Supplementary Methods for details of calculation.

### Sampling the parameter space

We randomly selected 10,000 points from the parameter space corresponding to the concentrations of 11 external metabolites and the G3P and pyruvate concentrations. Each parameter was sampled logarithmically over a range covering several orders of magnitude above and below its typical physiological concentrations (see Supplementary Table 4 for details). For each of these points, we calculated the optimized flux *J*_*i*_ of each candidate pathway as detailed above, and computed the CF of path *i* as CF_*i*_=*J*_*i*_/max{*J*_*k*_}, by dividing its flux by the highest flux obtained across all pathways at the given point in parameter space.

### Robustness of our results to small free energy changes

Using the group contribution method, the typical error in our calculation of the free energy of formation Δ_*f*_*G* for a given molecule is a few kJ mol^−1^ (see Supplementary Methods for details). To check the robustness of our results to such errors, our entire analysis was repeated using Δ_*f*_*G* values computed using different sets of training molecules, consisting of 80% of the molecules from the original training set, chosen at random. The qualitative results using such networks were identical from those obtained from the full set of training compounds. For example, the top 25 glycolytic pathways obtained from the reduced set contained 23 out of the 25 pathways from the original analysis.

## Additional information

**How to cite this article:** Court, S. J. *et al*. Lower glycolysis carries a higher flux than any biochemically possible alternative. *Nat. Commun.* 6:8427 doi: 10.1038/ncomms9427 (2015).

## Supplementary Material

Supplementary Information Supplementary Figures 1-7, Supplementary Tables 1-4, Supplementary Notes 1-2, Supplementary Discussion, Supplementary Methods and Supplementary References

Supplementary Data Set 1 Glycolytic pathways from Figure 2b in main text. Columns are: EC class, standard free energy, reaction equation.

Supplementary Data Set 2 Gluconeogenic pathways from Figure 2d in main text. Columns are: EC class, standard free energy, reaction equation.

Supplementary Data Set 3 Glycolytic pathways (top 100) from comparison in which no constraints are placed on the intermediate metabolites. Columns are: EC class, standard free energy, reaction equation.

Supplementary Data Set 4 Glycolytic pathways from Supplementary Fig. 5. Analysis uses the extended range for feasible metabolite concentrations, i.e. 1 nM to 500 mM. Columns are: EC class, standard free energy, reaction equation.

Supplementary Data Set 5 Gluconeogenic pathways from Supplementary Fig. 5. Analysis uses the extended range for feasible metabolite concentrations, i.e. 1 nM to 500 mM. Columns are: EC class, standard free energy, reaction equation.

Supplementary Data Set 6 Glycolytic pathways from Supplementary Fig. 6, i.e. pathways contain only compounds found in the KEGG COMPOUND database. Columns are: EC class, standard free energy, reaction equation.

## Figures and Tables

**Table 1:** The set of enzymatic reaction types used in generating the network of reactions.

**EC class**		**Example reaction**
1.1.1	Oxidation	CH_3_-CH_2_(OH)+NAD^+^⇌CH_3_-CHO+NADH
1.2.1		phosphorylation	CH_3_-CHO+NAD^+^+Pi⇌CH_3_-COp+NADH
1.3.1			COOH-CH_2_-CH_2_-COOH+NAD^+^⇌COOH-CH=CH-COOH+NADH
1.4.1		Deamination	COOH-CH(NH_2_)-CH_3_+NAD^+^+H_2_O⇌COOH-CO-CH_3_+NADH+NH_3_
2.6.1	Transamination	COOH-CH(NH_2_)-CH_3_+2-oxoglutarate⇌COOH-CO-CH_3_+glutamate	
2.7.1	Phosphate transfer	CHO-CH_2_(OH)+ATP⇌CHO-CH_2_p+ADP	
2.7.2		CH_3_-COOH+ATP⇌CH_3_-COp+ADP	
2.7.9		CH_3_-CO-COOH+ATP+H_2_O/Pi⇌CH_2_=Cp-COOH+AMP+Pi/PPi	
3.1.3	Hydrolysis	CH_3_-CH_2_p+H_2_O⇌CH_3_-CH_2_(OH)+Pi	
3.5.1			CH_3_-CO(NH_2_)+H_2_O⇌CH_3_-COOH+NH_3_
3.6.1			CH_3_-COp+H_2_O⇌CH_3_-COOH+Pi
4.1.1	Decarboxylation	CH_3_-CH(OH)-COOH+H_2_O⇌CH_3_-CH_2_(OH)+CO_2_(*aq*)	
4.2.1	Dehydration	CH_3_-CH(OH)-COOH⇌CH_2_=CH-COOH+H_2_O	
4.3.1	Ammonia-lyase	COOH-CH(NH_2_)-CH_3_⇌COOH-CH=CH_2_+NH_3_	
5.3.1	Isomerization	CH_3_-CO-CH_2_(OH)⇌CH_3_-CH(OH)-CHO	
5.3.2			COOH-CH_2_-CO-COOH⇌COOH-CH=C(OH)-COOH
5.4.2			CH_3_-CH(OH)-CH_2_p⇌CH_3_-CHp-CH_2_(OH)
5.4.3			COOH-CH(NH_2_)-CH_2_-CH_3_⇌COOH-CH_2_-CH(NH_2_)-CH_3_
6.3.1	ATP-driven amine ligase	R-COOH+ATP+NH_3_⇌R-CO(NH_2_)+ADP+Pi	
6.4.1	ATP-driven carboxylation	CH_3_-CH_2_(OH)+CO_2_(*aq*)+ATP⇌CH_3_-CH(OH)-COOH+ADP+Pi	

The set of reaction types included in our analysis, defined by the first three numbers of the EC classification. Although we show non-ionic formulae, all compounds are assumed to exist as an equilibrium mixture of protonated and deprotonated species in solution (Supplementary Methods). Phosphate groups -
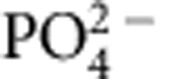
 are denoted by a ‘p'. Not all variants of each reaction class are listed here; for a complete list, see Supplementary Table 3.
