# Supplementary material for: Lower glycolysis carries a higher flux than any biochemically possible alternative
Source: Nat Commun. 2015 Sep 29;6:8427. doi: 10.1038/ncomms9427 (PMC4598745; doi:10.1038/ncomms9427)
Supplement: Supplementary Information — Supplementary Figures 1-7, Supplementary Tables 1-4, Supplementary Notes 1-2, Supplementary Discussion, Supplementary Methods and Supplementary References [file ncomms9427-s1.pdf]

1. SUPPLEMENTARY FIGURES

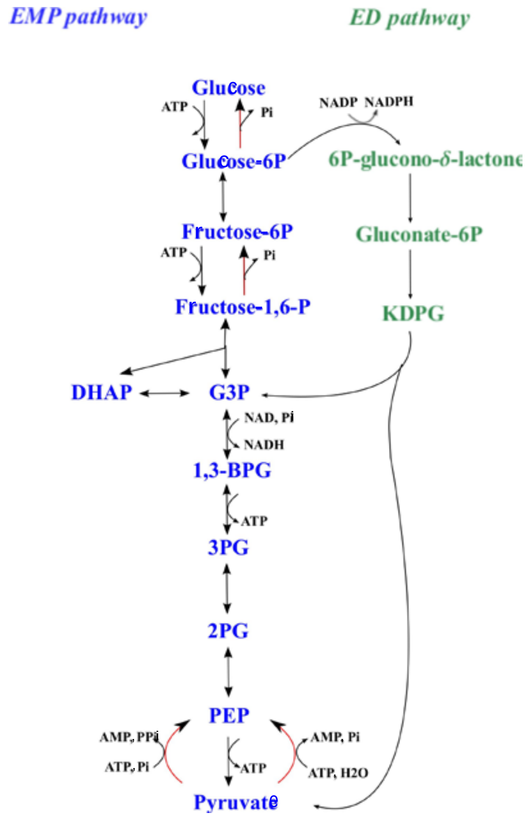

FIG. S 1. The Embden-Meyerhof-Parnas and Entner-Doudoroff glycolytic pathways. The red arrows indicate gluconeogenic reactions and KDPG denotes 2-keto-3-deoxy-6-phosphogluconate. These pathways share a common set of reactions in "lower glycolysis"; these reactions are known as the trunk pathway[1]. The pentose phosphate pathway[2] also feeds into this same trunk pathway. In prokaryotes, the trunk pathways in the glycolytic and gluconeogenic directions both have 5 steps and share almost the same set of reactions. The exception is the highly exergonic final step in the glycolytic direction: in glycolysis phosphoenolpyruvate (PEP) is transformed to pyruvate with the conversion of an ADP molecule to ATP via pyruvate kinase (EC 2.7.1.40); in gluconeogenesis two different solutions exist to overcome this energetic barrier. The most prevalent[1] is the phosphoenolpyruvate synthetase reaction (pps, EC 2.7.9.2) which converts pyruvate to phosphoenolpyruvate and couples with the conversion of ATP and water to AMP and inorganic phosphate. Alternatively, some organisms use pyruvate, phosphate dikinase (ppdk, EC 2.7.9.1) which couples the reaction to the conversion of ATP and inorganic phosphate to AMP and pyrophosphate. Under the conditions assumed in this work ($T = 25\ ^0$C, pH = 7.0, and ionic strength $I = 0.2$ M) and assuming standard concentrations of 1 M, the pps reaction has a free energy change of $\Delta_r G^0 = -5.0$ kJ mol^{-1} while ppdk is less thermodynamically favourable with $\Delta_r G^0 = +17.7$ kJ mol^{-1}. However changes in the concentrations of the external metabolites, in particular inorganic phosphate [Pi] and pyrophosphate [PPi], can change the relative favourability of these reactions (see Fig. 3 main text).

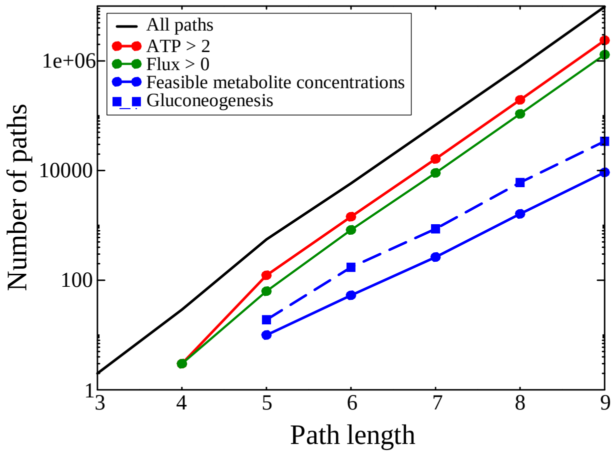

FIG. S 2. The number of pathways connecting G3P and pyruvate in our network increases exponentially as a function of the path length (number of reactions). We enumerate pathways between G3P and pyruvate in our network using the depth-first search algorithm. The number of pathways found increases approximately exponentially with pathway length (black line). Applying realistic thermodynamic and biophysical restrictions greatly reduces the number of feasible pathways. In the glycolytic direction, the red line shows the number of pathways that produce at least 2 ATP molecules, the green line shows the number of pathways that produce at least 2 ATP molecules and carry a positive flux under typical physiological conditions (as defined by the set of parameters below) and the blue line shows the number of pathways that produce at least 2 ATP molecules, carry a positive flux, and have intermediate concentrations between 0.1 μM and 10 mM. The dashed blue line shows the number of gluconeogenic paths which carry a positive flux under these typical physiological conditions and also have intermediate concentrations within the allowed range. The "typical physiological conditions" discussed, correspond to (all concentrations in mM) [G3P]=[PYR]=0.01, [ATP]=5.0, [ADP]=1.0, [AMP]=0.1, [NAD^+]=1.0, [NADH]=0.1, [Pi]=[PPi]=1.0 and [CO_2]=0.1, [NH_3]=0.01, [GLUT]=20.0 and [OXO]=1.0.

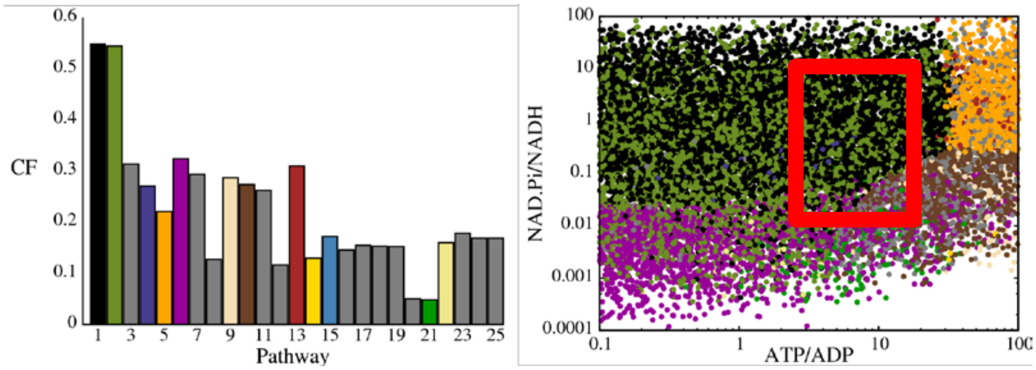

FIG. S 3. Comparison of glycolytic trunk pathways using reversible Michaelis-Menten kinetics. The colours and parameter sampling are the same as in Figure 2 of main text. All enzymes are assumed identical, with parameters $V_f = 10^{-3}$ M s^{-1}, $K_S = K_P = 10^{-5}$ M (see Supplementary Methods). Although the ordering of the pathways does differ from that when assuming perfect enzymes, the real trunk pathway still tends to perform best under typical physiological conditions (red box).

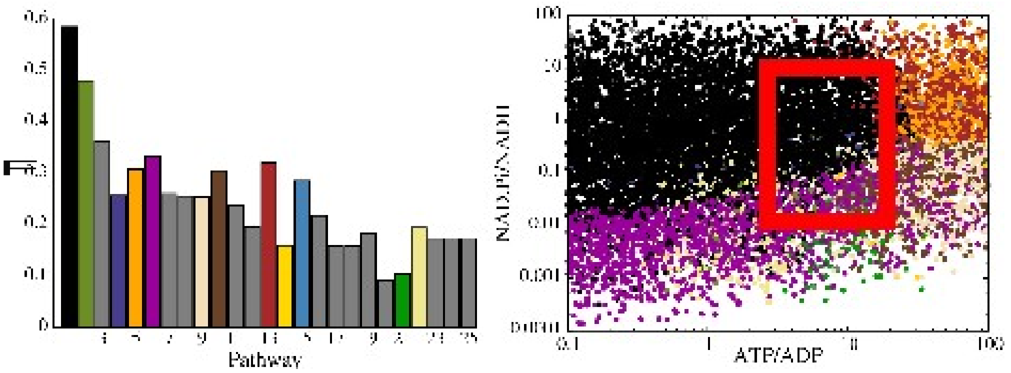

FlO. S 4. Further oomp ison of glycolytic b-unk pathways using l'eVE:lsible Mid\aelis-Menten kinetics. Again, all enzymes are assumed identioal, with parameters now $Vt = 10^{-3}$ M s–1, $Ks = 10^{-6}$ M,.f(p = 10^{-3} M (see Supplementary Methods). Colour oodingis same as in Fig. 2 of main text and 1'Ed boxindioates typioal physioloQ.cal conditions.

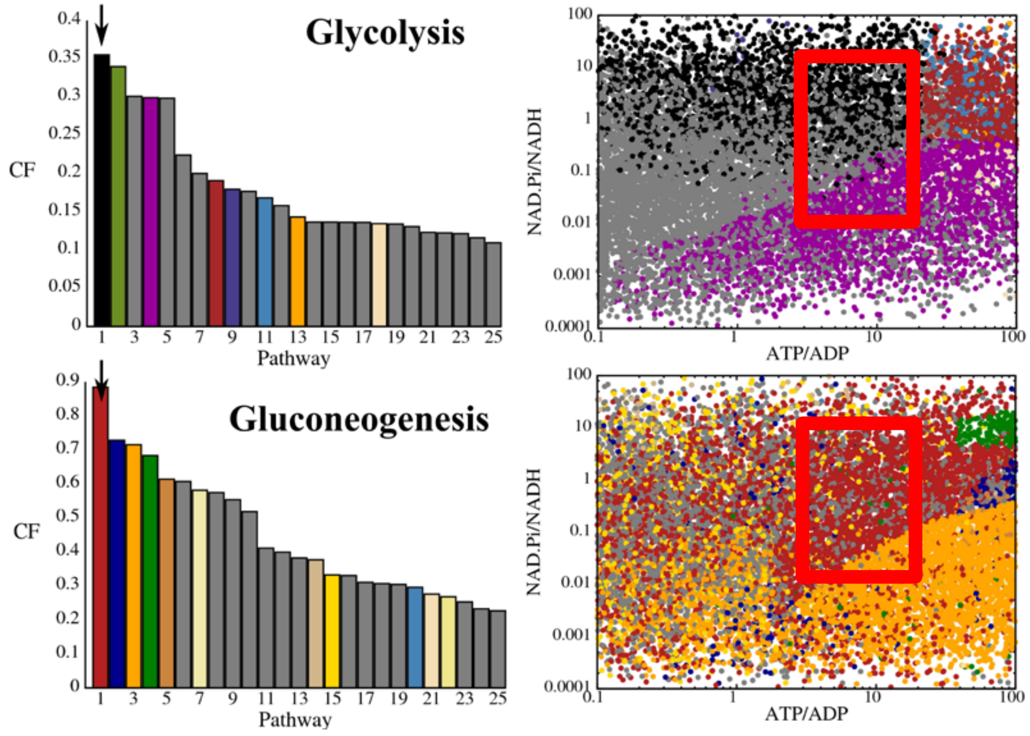

FIG. S 5. Comparative flux, CF, for each pathway averaged over all parameter space (left) and best performing pathway in different regions of parameter space (right) where the allowed range of metabolite concentrations has been increased to 1 nM to 500 mM. We find a similar outcome to the case of the smaller range used in the main text (Fig. 2): the real pathways (shown by arrows in the left panels) outperform the alternatives in the physiological region of parameter space (right panels). The colour coding is as in Fig. 2 of the main text. Glycolytic pathways are shown on the top row, gluconeogenic on the bottom row, (see Supplementary Data 4 and 5 for pathway structures).

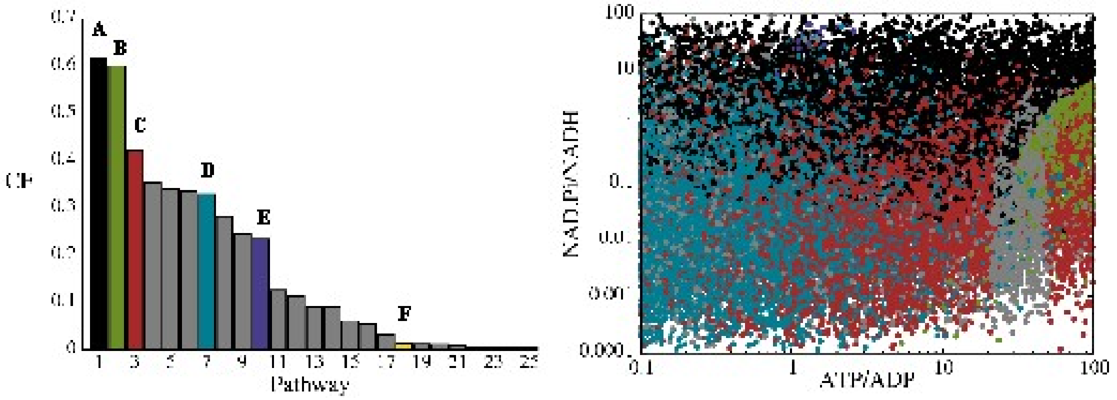

FIG. S 6. Comparison of alternative glyoolytic pathways generated that use only oompounds found in KEOO COMPOUND database. SeeSupplementa<y Note 2 arid Supplementa<y Data6 ror pathw,.ydetails. Flux oalculat.ion and parameter ranges as in Fig. s:i.

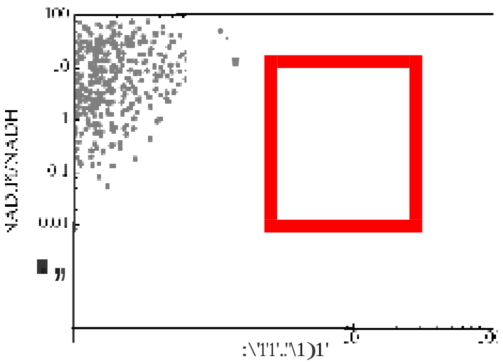

FIG. S 7. Length 6 pathways producing g ATP molecules from oonversion of GaP to pyruvate are not thermodynamioally feasible under typical physioloQ.cal oonditions (red rectangle). Enumerating pathways of length 6 yields 22a pat.hws potentially oapable of generating g ATP molecules, however these pathways only beoorne feasible for low (ATP(/(ATP(and hi&tl (NAD[(Pi(/(NADH(ratios, as shown by the grey dots.

2. SUPPLEMENTARY TABLES

ATP	ADP	AMP
NAD^+	NADH	CO_2 (*aq*)
Pi	PPi	H_2O
NH_3 (*aq*)	GLUT	2-OXO

TABLE S 1. The set of external metabolites used in our analysis. Glutamate (GLUT) and 2-oxoglutarate (2-OXO) are treated as external metabolites, acting as amino-group donor and acceptor respectively; Pi and PPi are orthophosphate and pyrophosphate, respectively.

**$-CH_3$**	**$-CH_2(OH)$**	**-COOH**	**-CHO**	**$-CH_2p$**
**-COp**	**$=CH_2$**	**=CH(OH)**	**=CO**	**=CHp**
**$-CH_2(NH_2)$**	**$-CO(NH_2)$**	-CH(OH)-	-CO-	-CHp-
-CH=	-C(OH)=	-Cp=	$-CH_2-$	$-CH(NH_2)-$

TABLE S 2. The set of groups used to construct the internal metabolites in our network. Groups in bold indicate those which must be present at the ends of a molecule to prevent “dangling bonds”. Note that the phosphate group “p” ($-OPO_3^{2-}$) and carboxyl group “COOH” both denote a mixture of protonated and deprotonated forms. This is taken into account in the calculation of free energies of formation (see Supplementary Methods).

**Reaction type**	**EC**	**Generalised reaction(s)**
oxidation	1.1.1	R-CH(OH)-R^t + NAD ;-- R-CO-R^t + NADH
(& carboxylation)	1.1.1	R-CH(OH)-r'-COOH + NAD ;-- R-CO-r'-H + CO_2(aq) + NADH
	1.2.1	R-CHO + NAD + H_2O ;-- R-COOH + NADH
(& phosphorylation)	1.2.1	R-CHO + NAD + Pi ;-- R-COp + NADH
	1.3.1	R-CH_2-CH_2-R'+ NAD ;-- R-CH=CH-R' + NADH
	1.3.1	R-CH(OH)-CH(OH)-R'+ NAD ;-- R-C(OH)=C(OH)-R' + NADH
(& deamination)	1.4.1	COOH-CH(NH_2)-R + NAD + H_2O ;-- COOH-CO-R + NADH + H^+
amino transfer	2.6.1	COOH-R-CO-R' + GLUT ;-- COOH-R-CH(NH_2)-R' + 2-OXO
	2.7.1	R-OH + ATP ;-- R-p + ADP
	2.7.1	R=Cp-R^t + ADP ;-- RH-CO-R^t + ATP
phosphate transfer	2.7.2	R-COOH + ATP ;-- R-COp + ADP
	2.7.9	R=CP-R^t + AMP + Pi ;-- R-CO-R^t + ATP + H_2O
	2.7.9	R=Cp-R^t + AMP + PPi ;-- R-CO-R^t + ATP + Pi
hydrolysis	3.1.3	R-p + H_2O ;-- R-OH + Pi
	3.5.1	R-CO(NH_2) + H_2O ;-- R-COOH + NH_3
	3.6.1	R-COp + H_2O ;-- R-COOH + Pi
decarboxylation	4.1.1	R-COOH + H_2O ;-- R-H + CO_2(aq)
(& phosphorylation)	4.1.1	COOH-R-CO-R' + Pi ;-- R=Cp-R' + CO_2(aq)
	4.1.1	COOH-R-CO-R' + ATP ;-- R=Cp-R' + ADP + CO_2(aq)
dehydration	4.2.1	R(H)-(OH)R^t ;-- R=R^t + H_2O
	4.2.1	R(OH)-(OH)R' ;-- R(=O)-R' + H_2O
	4.2.1	R-CH(OH)-COOH ;-- R(=O)-CHO + H_2O
deamination	4.3.1	COOH-CH(NH_2)-(H)R ;-- COOH-CH=R + NH_3
	4.3.1	COOH-CH(NH_2)-CH(OH)-R ;-- COOH-CO-CH_2-R + NH_3
	5.3.1	R-CO-CH_2(OH) ;-- R-CH(OH)-CHO
	5.3.2	R=C(OH)-R^t ;-- RH-CO-R^t
isomerisation	5.4.2	R-CH(OH)-CH_2p ;-- R-CHp-CH_2(OH)
	5.4.2	R=C(OH)-CH_2p ;-- R=Cp-CH_2(OH)
	5.4.2	R-CHp-COOH ;-- R-CH(OH)-COp
	5.4.2	R=Cp-COOH ;-- R=C(OH)-COp
	5.4.3	R-CH(NH_2)-CH_2-R' ;-- R-CH_2-CH(NH_2)-R'
	5.4.3	R-CH(NH_2)-CO-R ;-- R-CO-CH(NH_2)-R'
ATP-driven amination	6.3.1	R-COOH + ATP + NH_3 ;-- R-CO(NH_2) + ADP + Pi
ATP-driven carboxylation	6.4.1	RH-CO-COOH + CO_2(aq) + ATP ;-- COOH-R-CO-COOH + ADP + Pi

TABLE S 3. The set of reaction types included in our network, with all external metabolite couplings. Here, R and R^t denote arbitary chemical groups (not necessarily linear chains) and can represent a single H atom. Pi denotes orthophosphate; PPi denotes pyrophosphate, and p denotes the phosphate group of a phosphorylated substrate. All compounds are assumed to exist as an equilibrium mixture of protonated and deprotonated species in solution.

Parameter	Range sampled
[source]	1μM to 1 mM
[source]/[product]	0.01 to 100
[ATP]/[ADP]	0.1 to 100
[NAD]/[NADH]	0.1 to 1000
[AMP]	0.01 to 0.1 mM
[Pi]	1.0 to 100 mM
[PPi]	0.1 to 10 mM
[CO_2]	1.0 μM to 0.1 mM
[NH_3]	1.0 μM to 0.1 mM
[GLUT]	1.0 to 100 mM
[2-OXO]	0.1 to 10 mM

TABLE S 4. Parameters sampled independently in our analysis and the ranges over which they were sampled. Ranges chosen are representative of those found in literature (see[3–5] and references therein). The source and product concentrations correspond to G3P and PYR in the glycolytic direction and vice versa in the gluconeogenic direction.

3. SUPPLEMENTARY NOTES

Note 1: Expanding the range of feasible intermediate metabolite concentrations

Entirely removing the restrictions on the steady state metabolite concentrations strongly alters the outcome of the analysis, but we find that imposing any sensible limits results in the natural trunk pathways performing best compared to the alternatives. There are good reasons for imposing limits on the intermediate metabolite concentrations. High metabolite concentrations can produce osmotic stress and increase the rate of disadvantageous spontaneous reactions, such as the non-enzymatic conversion of dihydroxyacetone to the toxic intermediate methylglyoxal[6]. On the other hand, metabolite concentrations that are too low are undesirable due to vanishing reaction fluxes. Experimentally it has been observed that metabolite concentrations can span several orders of magnitude, with most concentrations falling between 0.1 μM and 100 mM and the total intracellular metabolite pool, measured in *E. coli*, being around 300 mM[3].

In the main text we restricted all intermediate metabolite concentrations to lie between 10^{-7} M and 10^{-2} M in agreement with experimental findings and previous works[3,4,7]. These limits may be too restrictive, since in reality the trunk pathway does not exist in isolation, but other metabolic pathways branch out from and feed into it. In addition, the spatial structure of a cell, as well as phenomena such as substrate channelling, could feasibly lead to local metabolite concentrations that fall well outside this range.

Here we investigate the effect of allowing a much wider range of intermediate metabolite concentrations, from 1 nM to 500 mM. The lower bound here roughly corresponds to a concentration of 1 molecule per *E.coli* cell and the upper limit corresponds to about five times the highest concentration measured in *E. coli*[3]. Although such values are not perhaps observed in nature, they are not obviously unphysical, and could occur at least on a local scale. Figure S5 show the same comparison as in the main text, Fig. 2, but with these updated concentration ranges. Reassuringly, although the pathways present in the top 25 are altered, the real trunk pathways still perform best in the physiological region. In the case of glycolysis (top panels) 13 of the top 25 pathways from the main text Fig. 2b are present in this top 25. For gluconeogenesis (bottom panels) 18 of the pathways from Fig. 2d of the main text are present (see Supplementary Data 4 and 5).

Note 2: Alternative pathways utilizing compounds found in KEGG database

Pathway comparison

In Fig. 4 of the main text we discuss some of the alternative glycolytic pathways identified in our analysis that contain only metabolites found in the KEGG COMPOUND database. This database contains all known small molecules, biopolymers, and other chemical substances that are relevant to biological systems. Figure S6 shows a similar comparison to Fig. 2 of the main text, when only pathways using known metabolites are considered (see also Supplementary Data 6). The colours and labelling are the same as those in Fig. 4 main text.

Putative pathways contained in KEGG

Figure 4 of the main text depicted 3 alternative glycolytic pathways – B, D and E – for which all enzymes are known to occur in nature. Whether such pathways are ever realised however, depends on whether or not all enzymes occur in the same organism, and if they are ever coexpressed. The KEGG ORGANISM database contains about 3,400 complete genomes and assigns enzymes to each organism based on homology. Using this database, we checked whether we could find any organisms in which all the enzymes of a given putative glycolytic pathway were known to exist.

One complication is that certain reactions can be performed by mutiple enzymes. For instance, the first step of Paths B and E, glyceraldehyde 3-phosphate dehydrogenase (phosphorylating) labelled 1.2.1.12 in Fig. 4, couples with NAD^+. However, two other characterized enzymes are also associated with this reaction, with different couplings: EC 1.2.1.13 couples with NADP, while 1.2.1.59 couples with both NAD^+ and NADP with equal efficiency. We have deemed an organism capable of this step if it contains 1 or more of any of these 3 enzymes. Similarily, EC 5.4.1.11 (2,3-BPG dependent) can also be performed by 5.4.1.12 (2,3-BPG independent) as well as EC 5.4.2.4; EC 1.2.1.9 (coupling to $NADP^+$) can also be performed by 1.2.7.6 (utilizing oxidized ferredoxin); EC 4.1.1.49 (producing ATP) can be replaced with 4.1.1.32 (producing GTP).

Taking account of these possible substitutions, searching the KEGG ORGANISM database results in 348 organisms whose genomes encode all the enzymes for Path B, 160 organisms which can potentially produce all of the enzymes for Path D and 982 which can potentially express Path E. This analysis only considers whether the genome sequence of a given organism encodes all enzymes needed for a pathway; it is also of course necessary for those enzymes to be coexpressed. Further work might consider whether these organisms express the relevant pathways; however available database information on gene expression is limited to a small number of organisms.

In the present work we have restricted our analysis to conditions of pH $= 7$ and T $= 25^0$C. It might be that different environmental conditions, specified by pH or temperature, play a role in determining the feasibility of the various pathways identified. For example, reducing the pH from 7 to 6 makes the creation of 2 ATP molecules from PEP (as in path D, Fig. 4 main text) more thermodynamically favourable by about 5 kJ mol^{-1}. Future work will investigate such environmental dependencies in full detail.

4. SUPPLEMENTARY DISCUSSION

Although the trunk pathway is highly ubiquitous in nature, some variants have been characterized. Here we discuss these variants and compare them to the alternative pathways that emerge from our analysis. We also assess what evolutionary goal functions (if any) these variant pathways might be optimizing.

Glycolytic variants

One variation which can be found in plants and certain photosynthetic organisms[8,9] involves the bypass of 1,3-BPG through the essentially irreversible direct oxidation of G3P to 3PG as seen in the first step of glycolytic path D (Fig. 4 main text, EC 1.2.1.9). The use of this bypass results in no ATP production in glycolysis, but it is possible that organisms which do not rely on the degradation of sugars for energy, e.g. phototrophs, can sacrifice the glycolytic generation of ATP so as to attain a greater flux of creation of biosynthetic intermediates. This shortcut is also present in some bacteria and archaea[1,10,11].

Hyperthermophilic bacteria and archaea are known to use another distinct variation. At high temperatures metabolite stability, especially for phosphorylated compounds, can be very low (Section 4) with 1,3-BPG being one of the least stable metabolites[12]. The nonphosphorylating variant of the Entner-Doudoroff pathway present in hyperthermophiles almost entirely avoids phosphorylated intermediates[11]. The unphosphorylated version of KDPG (KDG) is instead cleaved to form glyceraldehyde and pyruvate, with glyceraldehyde being dehydrated to glycerate before being phosphorylated to 2-PG and re-entering the trunk pathway. Again, this path results in no ATP production and so its existence must be explained by other principles.

Finally, another atypical glycolytic pathway has been found in the bacterium *Clostridium thermocellum*[13]. This organism was found to lack a pyruvate kinase enzyme for the glycolytic conversion of PEP to pyruvate. Ref.[13] determined that the most likely pathway involved the conversion of PEP to oxaloacetate, oxaloacetate to malate and finally malate to pyruvate. This pathway involves an additional two reactions compared to the usual pathway. This pathway is particularly interesting since it can actually be used to generate one more NADPH than the more common trunk pathway, and generates GTP as well as ATP. It would be interesting to see if our analysis selects this pathway when considering a different goal function that incorporates the production of an additional reducing agent.

Gluconeogenic variants

In this work our analysis focused on prokaryotic metabolism. In prokaryotes, the gluconeogenic trunk pathway has 5 steps, with the initial step being the direct transformation of pyruvate to PEP coupled to the conversion of ATP to AMP (via the pps or ppdk enzymes). The initial steps of the textbook picture of eukaryotic gluconeogenesis are slightly different. Here, pyruvate is converted to PEP in two steps: pyruvate carboxylase (EC 6.4.1.1) converts pyruvate to the 4-carbon molecule oxaloacetate, which is then simultaneously de-carboxylated and phosphorylated to form PEP by phosphoenolpyruvate carboxykinase (EC 4.1.1.32). The first step is driven by the dephosphorylation of ATP to ADP while the second step is driven by the dephosphorylation of GTP. This pathway

therefore has 6 steps and in total consumes 3 “energy units” (2 ATPs are converted to ADP while 1 GTP is converted to GDP). When we repeat our analysis in the absence of dikinase reactions (i.e. not allowing the conversion of ATP to AMP), we find that 5-step gluconeogenic pathways are no longer thermodynamically feasible under typical physiological conditions since biochemically they are only able to consume 2 ATP molecules (both coupling to ATP → ADP). It is not clear why this pathway seems to be predominantly present in eukaryotic organisms, especially higher animals which are often lack both the pps and ppdk genes. One possible benefit is that it serves to compartmentalize glycolysis and gluconeogenesis and facilitate the transport of NADH between the cytoplasm and mitochondria.

Are the chemical compounds generated by our network compatible with life?

Many of the chemical compounds generated in our study have not been characterized and may never have occurred in biology. This raises the question of whether they would be stable in a biological cell, and whether they might in fact be toxic to a cell.

Stability

When considering the stability of our putative metabolites, it is important to bear in mind that some of the metabolites actually used in nature are relatively unstable in solution, spontaneously degrading into potentially unwanted side products, especially at high temperatures. For example, for the thermophile *Sulfolobus solfataricus*[12] it has been shown that during gluconeogenesis at a temperature of 70^oC, as much as 50% of the total carbon flow could be lost due to thermal degradation.

Some of the compounds used in our alternative pathways do nevertheless raise concerns as to stability. In particular, a small number of pathways contain enol compounds (containing a hydroxyl group attached to a C=C double bond), compounds that commonly undergo a spontaneous tautomerization to the more stable keto-form. For instance, 4 of the 25 glycolytic pathways of Fig. 2 in the main text contain an enol. Future work could automatically replace any enol with its keto tautomer so as not to assign any enzyme reaction kinetics to this likely spontaneous reaction. However, enzymes do exist that catalyze these tauomerizations (EC class 5.3.2), and it may not be obvious which form is most stable for a given set of conditions. A similar argument can be made for ketenes (C=C double bond in which one is a carbonyl group) since these also spontaneously convert, in a reaction with water, to a carboxylic acid[8]. However, none of the pathways in the present study contain a ketene intermediate.

Toxicity

With regard to toxicity, while it cannot be ruled out that some of our compounds are toxic, most of them are chemically similar enough to molecules known to be utilized by living organisms to make them plausible alternatives. For example, to a greater or lesser extent, all carbonyl groups present in aldehydes and ketones react with DNA, proteins and other biological macromolecules, inducing mutations and inactivating enzymes[8]. Different carbonyl groups can exhibit a range of activities however, and carbonyl-containing compounds such as glucose, G3P and DHAP clearly do not

impose any serious problems for life, though they do have a measurable toxic effect[14]. Metabolites that contain two carbonyl groups, either on adjacent or next-adjacent carbons, tend to show very high reactivity and can cause damage even at low levels. An example of this is methylglyoxal, the toxic product of the uncatalyzed dephosphorylation of DHAP. But despite its high toxicity, this metabolite does appear in real cells in the pathway known as the methyglyoxal shunt. For these reasons we do not attempt to exclude any compounds from the network on the grounds of toxicity. Future work could however address this question in more detail.

5. SUPPLEMENTARY METHODS Construction of our

network of all possible chemical reactions *Metabolites*

As discussed in the main text, we restrict our list of internal metabolites to unbranched, aliphatic 2, 3 and 4-carbon CHOPN molecules (i.e. those containing only carbon, hydrogen, oxygen, nitrogen and phosphorus atoms, with the phosphorus being in the form of phosphate groups). We exclude hydrocarbons (i.e. molecules with no oxygen atoms), C≡C triple bonds and esters. We also neglect chirality. We consider only molecules which are charged, i.e. contain at least one carboxyl or phosphate group; this is because nonpolar molecules can have very high membrane permeability[8], making them prone to leaking out of the cell. In our computer program, each internal metabolite is constructed as a linear assembly of the groups shown in Table S2. Note that we do not allow these groups (containing a single carbon atom) to contain more than one hydroxyl or phosphate group on the grounds of stability. As an example, the important metabolite pyruvate is produced by combining, in order, the three groups CH_3-CO-COOH. The full list of internal metabolites is generated systematically by combining these groups in all possible permutations, eliminating any molecules which do not satisfy the above restrictions.

Many of the reactions in our network involve cofactors such as ATP, ADP, NAD and NADH. These are classified as *external metabolites* in our analysis, and their concentrations define the intracellular conditions. The external metabolites included in our analysis are listed in Table S1. We assume that, in solution, these compounds exist as a mixture of different dissociated forms; for example ATP exists in cells as a mixture of ATP^{4-}, $HATP^{3-}$, H_2ATP^{2-} etc, while "CO_2 (aq)" in our table represents H_2CO_3 and its various forms HCO_3^-, CO_3^{2-}, etc. This is taken into account in the calculation of the free energy as described in Section 5.

Reactions

Our program systematically constructs all possible reactions connecting pairs of internal metabolites, consistent with the 19 EC classes listed in Table 1 of the main text. The EC classification[15] consists of four numbers that provide increasingly detailed information about the action of an enzyme. For instance EC 5 corresponds to isomerases, EC 5.4 to intramolecular transferases and EC 5.4.2 to phosphotransferases (isomerases that transfer a phosphoryl group from one carbon to another on the same molecule). The fourth number indicates the specific substrates and products. The reaction classes in our network are described by just the first 3 EC numbers, since they describe mechanistically equivalent chemical transformations, carried out on different chemical substrates. For each reaction class, we include all known couplings to the external metabolites. This means that a given pair of internal metabolites may be connected by more than one reaction (edge) in our network, e.g. the three different reactions coupling PEP and pyruvate in Fig. S1. Reactions coupling to different external metabolites will involve different free energy changes and will be affected by the cellular environment in different ways. Table S3 provides a complete list of the reaction types included in our analysis. In total our network contains 1477 metabolites and 7940 reactions.

Estimating reaction free energies

We calculate the biological standard free energy change $\Delta_r G'$ (denoted by a prime; defined under conditions with T= $25\,^oC$, pH = 7.0, I = 0.2 M, and with all metabolite concentrations set to 1 M) associated with a given reaction in our network by subtracting the free energy of formation of the reactants from that of the products, including the external metabolites (cofactors):

$$\Delta_r G' = \sum_{i \in \{\text{products}\}} \Delta_f G'_i - \sum_{j \in \{\text{reactants}\}} \Delta_f G'_j \,. \tag{1}$$

This requires us to know the free energies of formation $\Delta_f G'$ for all the molecules in the network. However, for many of the compounds, no experimental value for $\Delta_f G'$ is available. In these cases we estimate $\Delta_f G'$ using a group contribution method based on Refs.[16–19]. The basic idea behind the group contribution method is that any molecule can be split into a number of functional groups, each making a contribution to the total free energy of formation of the molecule. Assuming that all molecules are built from a linear combination of the functional groups $\{g_i\}$ listed in Table S2, we write their $\Delta_f G'$ as

$$\Delta_f G' = E_0 + \sum_j E_1(g_j) + \sum_{<j,k>} E_2(g_j, g_k), \tag{2}$$

where E_0 is a constant (which is the same for all molecules), $E_1(g_j)$ is the contribution of group g_j and $E_2(g_j, g_k)$ is a small correction due to neighbouring group-group interactions. Alberty[20] has listed standard Gibbs free energies of formation for around 200 compounds of biological interest, 64 of which are linear CHOPN molecules as in our network. We obtained the values of E_0, the vector E_1 and matrix E_2 by performing a least-squares fit to this set of 64 data points. We also account for the fact that chemicals in aqueous solution can exist as a mixture of dissociated species[21,22]. The free energy of formation $\Delta_f G'$ of compound A at a given pH and ionic strength I can be written as[21]:

$$\Delta_f G' = \Delta_f G^{\text{pH},I}_{(A)} = -RT \ln \left(\sum_i \exp\left(-\frac{\Delta_f G^0_i}{RT} + N_H(i) \ln 10^{-\text{pH}} - \frac{2.91482(Z_i^2 - N_H(i)) I^{1/2}}{1 + 1.6 I^{1/2}} \right) \right), \tag{3}$$

where the index i runs over all differently protonated forms of A (H_nA, $H_{n-1}A^-$, $H_{n-2}A^{2-}$, etc.), Z_i is the charge of the form i, $N_H(i)$ is the total number of hydrogen atoms of i, and $\Delta_f G^0_i$ is the standard free energy of formation of that particular form (25 °C, pH = 0, 1 M concentrations, and zero ionic strength $I = 0$ M). The sum over i comes from the entropy of mixing of different forms of A. The term proportional to $N_H(i)$ accounts for changes in concentrations of different forms for non-zero pH, and the last term gives a correction for non-zero ionic strength ($I > 0$, due to the presence of ions such as Na^+, Cl^- etc.). We use Eq. (3) to calculate free energies of the training molecules used to obtain parameters for the group contribution method. Note that, since we use Eq.(3) to calculate the free energies of the training molecules, the parameters E_0, E_1, and E_2 as determined by us are valid only for the specific set of conditions, and hence the free energies of our CHOPN molecules are also valid only for this specific set of conditions. The thermodynamic data available allows for Eq. (3) to be used for pH values between 5 and 9, and ionic strengths up to I = 0.25 M.

For our 64-compound training set, we find that the error – the square root of the variance of absolute differences between the experimental $\Delta_f G^I$ values and those calculated using the group contribution method – to be 3.84 kJ mol^{-1}. Although these errors in $\Delta_f G^I$ could, in principle, combine to produce large errors in the reaction free energies, we find that in practice this is not the case. Performing our full analysis using networks trained on various subsets of the 64 training compounds demonstrates that our conclusions are robust. For instance, training the group contribution method on random samples of 80% of the compounds in the full training set can alter the free energies of formation by a few kJ mol^{-1} but does not qualitatively change the results of our analysis.

E0, E1 and E2 values obtained from least-squares fitting

Below are the values for E_0, E_1 and E_2 obtained from the least-squares fitting to the thermodynamic data of Alberty[20] :

$E_0 = -405.037$ kJ mol^{-1};

E_1: vector of group contributions (kJ mol^{-1}). The first column shows each molecular group, with the second column showing its contribution to the free energy of formation of a molecule;

E_2: matrix of corrections arising from group-group interactions (kJ mol^{-1}). First row and first column indicate the molecular groups.

$E1 =$

-CH3	307.787
-CH2(OH)	157.397
-COOH	−142.999
-CHO	121.208
-CH2p	−735.909
-COp	−1001.85
-CH2(NH2)	371.314
-CO(NH2)	107.454
=CH2	325.207
-CH2-	82.9466
=CH(OH)	140.302
-CH(OH)-	−72.9298
=CO	268.034
-CO-	−110.933
=CHp	−727.239
-CHp-	−951.415
-CH(NH2)-	155.24
-CH=	83.581
-C(OH)=	−99.4453
-Cp=	−966.986

$E2 =$

	-CH3	-CH2(OH)	-COOH	-CHO	-CH2p	-COp	-CH2(NH2)	-CO(NH2)	=CH2	-CH2-	=CH(OH)	-CH(OH)-	=CO	-CO-	=CHp	-CHp-	-CH(NH2)-	-CH=	-C(OH)=	-Cp=
-CH3	16.75	2.50948	−7.68032	−0.0940597	0	−8.06407	0	0	0	9.32333	0	−0.0610771	0	−7.26424	0	0	−2.04337	0	0	0
-CH2(OH)	2.50948	0	−18.9232	0	0	0	0	0	0	−2.46572	0	−1.97712	0	−0.739063	0	0.196125	8.47616	0	0	0
-COOH	−7.68032	−18.9232	14.0936	−1.81671	0	0	0.392249	0	0	−1.47864	0	5.36891	0	9.77311	0	0.196125	1.06492	0.13075	0.196125	0.196125
-CHO	−0.0940597	0	−1.81671	0	0	0	0	0	0	1.86562	0	5.30933	0	0	0	0	0	0	0	0
-CH2p	0	0	0	0	0	0	0	0	0	0	0	−3.74738	0	−1.89767	0	0	0	0	0	0
-COp	−8.06407	0	0	0	0	0	0	0	0	0	0	12.596	0	0	0	0	0	0	0	0
-CH2(NH2)	0	0	0.392249	0	0	0	0	0	0	0	0	0	0	0	0	0	0	0	0	0
-CO(NH2)	0	0	0	0	0	0	0	0	0	−0.0388611	0	0	0	0	0	0	0	0	0	0
-CH2-	9.32333	−2.46572	−1.47864	1.86562	0	0	0	−0.0388611	0	−1.55222	0	−5.10976	0	−5.09816	0	0	0.142301	0	0	0
=CH(OH)	0	0	0	0	0	0	0	0	0.392249	0	0	0	0	0	0	0	0	0	0	0
-CH(OH)-	−0.0610771	−1.97712	5.36891	5.30933	−3.74738	12.596	0	0	0	−5.10976	0	−0.624411	0	8.3408	0	0	−5.51018	0	0	0
=CO	0	0	0	0	0	0	0	0	0.392249	0	0	0	0	0	0	0	0	0	0	0
-CO-	−7.26424	−0.739063	9.77311	0	−1.89767	0	0	0	0	−5.09816	0	8.3408	0	0	0	0	0	0	0	0
=CHp	0	0	0	0	0	0	0	0	0.392249	0	0	0	0	0	0	0	0	0	0	0
-CHp-	0	0.196125	0.196125	0	0	0	0	0	0	0	0	0	0	0	0	0	0	0	0	0
-CH(NH2)-	−2.04337	8.47616	1.06492	0	0	0	0	0	0	0.142301	0	−5.51018	0	0	0	0	0	0	0	0
-CH=	0	0	0.13075	0	0	0	0	0	0	0	0	0	0	0	0	0	0	0.13075	0	0
-C(OH)=	0	0	0.196125	0	0	0	0	0	0.196125	0	0	0	0	0	0	0	0	0	0	0
-Cp=	0	0	0.196125	0	0	0	0	0	0.196125	0	0	0	0	0	0	0	0	0	0	0

Calculating the flux through a linear pathway using the perfect enzyme assumption

The results shown in the main text used flux calculations based on an assumption of perfect catalysis (although later in section 5 we show that our results are robust to the choice of kinetic model). This calculation is described in detail here.

To compute the flux carried by our candidate pathways we generalise the method of Heinrich et al.[23], extending it to the case where reactions include both internal and external metabolites. Let us consider an unbranched chain of n reactions with fixed concentrations of the initial substrate $[S_0]$ and final product $[S_n]$. For the simplest case, in which each reaction converts one substrate S_{i-1} into the next, S_i, and in which the flux v_i through reaction i obeys linear kinetics, we can write

$$v_i = k_i[S_{i-1}] - k_{-i}[S_i] = k_i \left([S_{i-1}] - \frac{[S_i]}{q_i} \right). \tag{4}$$

Here k_i and k_{i-1} are the first-order rate constants in the forward and backward directions and $q_i = e^{-\Delta_r G/RT} = k_i/k_{-i}$ is the thermodynamic equilibrium constant, where $\Delta_r G^I$ is the biological standard free energy change of the reaction as computed in our analysis using the group contribution method. We now generalise this to the case where external metabolites are involved. A generic reaction that converts internal metabolite S_{i-1} to S_i, with a coupled conversion of external metabolite e_{i-1} to e_i, can be expressed as

$$S_{i-1} + e_{i-1} \underset{\kappa_{-i}}{\overset{\kappa_i}{\rightleftharpoons}} S_i + e_i \tag{5}$$

By analogy with Eq. (4), we can write

$$v_i = \kappa_i[S_{i-1}][e_{i-1}] - \kappa_{-i}[S_i][e_i]. \tag{6}$$

Since we assume that all external metabolites are present at fixed concentrations (which determine the intracellular conditions), we can absorb the concentrations of the external metabolites into the rate constants, to obtain the pseudo first-order rate equation

$$v_i = K_i[S_{i-1}] - K_{-i}[S_i] = K_i \left([S_{i-1}] - \frac{[S_i]}{q_i^I} \right), \tag{7}$$

where $K_i = \kappa_i[e_{i-1}]$, $K_{i-1} = \kappa_{-i}[e_i]$, and $q_i^I = K_i/K_{-i} = \kappa_i[e_{i-1}]/\kappa_{-i}[e_i] = q_i \frac{[e_{i-1}]}{[e_i]} = \frac{[e_{i-1}]}{[e_i]} e^{-\Delta_r G/RT}$. Note that the q^I incorporates the shift in the equilibrium constant when taking into account the fixed external metabolite concentrations. We now consider a pathway consisting of a linear chain of such reactions. In steady state, the flux carried by each reaction is equal to the flux J through the pathway, *i.e.* $v_i = J$, for all i. Imposing this condition and rearranging Eq.(7) gives

$$[S_i] = q_i^I \left([S_{i-1}] - \frac{J}{K_i} \right). \tag{8}$$

Iterating this equation, starting from $i = 1$, results in an expression for the steady state concentrations of the intermediate metabolites, in terms of the rate constants, $\Delta_r G^I$ values, and the

concentration $[S_0]$ of the starting substrate:

$$[S_j] = [S_0] \prod_{i=1}^{j} q_i^I - J \sum_{i=1}^{j} \frac{1}{K_i} \prod_{m=i}^{j} q_m^I. \tag{9}$$

Setting $j = n$ in Eq. 9 and rearranging leads to an expression for the pathway flux J in terms of the rate constants, $\Delta_r G^I$ values, and the concentrations $[S_0]$ and $[S_n]$ of the initial and final substrates:

$$J = \frac{1}{D} \left([S_0] \prod_{i=1}^{n} q_i^I - [S_n] \right), \tag{10}$$

where

$$D = \sum_{i=1}^{n} \frac{1}{K_i} \prod_{m=i}^{n} q_m^I. \tag{11}$$

Perfect catalyst assumption

Eqs (10) and (11) require knowledge of the rate constants for all enzymes in the pathway (via the parameters K_i). For most of the reactions in our network, these parameters are not available. We therefore assume that all enzymes in our network behave as perfect catalysts - *i.e.* their reaction rate is limited only by the rate of diffusion of substrate to the enzyme. Because of this assumption, the flux obtained in our calculations for a given pathway can be thought of as representing the maximum possible flux sustainable by that pathway (note however that we show in section 5 that our results are robust to this assumption). Under this assumption the rate equation becomes[23–26]

$$v_i = \frac{k_d [E_i] \left([S_{i-1}] q_i - [S_i] \right)}{1 + q_i}, \tag{12}$$

where $[E_i]$ is the concentration of the enzyme catalyzing step i and k_d denotes the diffusion-limited rate constant. Complementary derivations of Eq.(12) have been presented by Heinrich et al.[24] and Albery et al.[25]. Here we derive Eq. 12 following the approach of Pettersson[26]. We consider a special case of the simple enzyme mechanism describing the reversible binding of a substrate S and product P to an enzyme E:

$$E + S \underset{k_{-1}}{\overset{k_1}{\rightleftharpoons}} ES \underset{k_{-2}}{\overset{k_2}{\rightleftharpoons}} EP \underset{k_{-3}}{\overset{k_3}{\rightleftharpoons}} E + P \tag{13}$$

The steady state of this process is given by[24]

$$v = \frac{E_0 (k_1 k_2 k_3 S - k_{-1} k_{-2} k_{-3} P)}{k_{-1}k_{-2} + k_{-1}k_3 + k_2 k_3 + (k_1 k_{-2} + k_1 k_3 + k_1 k_2) S + (k_{-1} k_{-3} + k_2 k_{-3} + k_{-2} k_{-3}) P}. \tag{14}$$

Following the approach of Pettersson[26], we consider an enzyme operating via scheme (13), in the absence of product ($P \to 0$ in Eq. 14). In this case, Eq. (14) reduces to a Michaelis-Menten rate equation:

$$v = \frac{E_0(k_1k_2k_3S)}{k_{-1}k_{-2} + k_{-1}k_3 + k_2k_3 + (k_1k_{-2} + k_1k_3 + k_1k_2)S} \equiv \frac{E_0 k_{cat} S}{K_M + S}, \tag{15}$$

yielding the following relationships between the physiological parameters and the microscopic rate constants

$$k_{cat} = \frac{k_2k_3}{k_{-2} + k_3 + k_2} \quad \text{and} \quad K_M = \frac{k_{-1}k_{-2} + k_{-1}k_3 + k_2k_3}{k_1(k_{-2} + k_3 + k_2)}. \tag{16}$$

We now assume that for enzymes operating in scheme (13), the substrate and product association rate constants k_1 and k_{-3} take the value $k_d = 10^9\ M^{-1}\,s^{-1}$, which is the rate constant for diffusion-controlled binding. We further assume, following Pettersson[26], that $k_2 \gg k_{-1}$ (Pettersson justifies this based on evolutionary considerations[26]). Writing

$$\frac{k_{cat}}{K_M} = \frac{k_1k_2k_3}{k_{-1}k_{-2} + k_{-1}k_3 + k_2k_3}, \tag{17}$$

and multiplying top and bottom by $1/k_1k_2k_3$, noting that the equilibrium constant of the reaction is $q = k_1k_2k_3/k_{-1}k_{-2}k_{-3}$, gives

$$\frac{k_{cat}}{K_M} = \frac{1}{\frac{1}{qk_{-3}} + \frac{k_{-1}}{k_1 k_2} + \frac{1}{k_1}}. \tag{18}$$

Applying the diffusion limit $k_1 = k_{-3} = k_d$ and assuming $k_2 \gg k_{-1}$, leads finally to the expression

$$\frac{k_{cat}}{K_M} \approx \frac{k_d}{1 + q^{-1}} = \frac{k_d q}{1 + q}. \tag{19}$$

At low substrate concentrations, the Michaelis-Menten rate equation Eq. (15) takes the linear form $v \approx (\frac{k_{cat}E_0}{K_M})S$. If we assume the reaction is operating in this linear regime, we can use this approximation to write

$$v = k_{forw}\, S - k_{back}\, P = \left(\frac{k_dE_0}{1 + q^{-1}}\right) S - \left(\frac{k_dE_0}{1 + q}\right) P, \tag{20}$$

$$v = k_dE_0 \left(\frac{Sq - P}{1 + q}\right), \tag{21}$$

which is Eqn. 12 for a perfect catalyst.

Expressing this as a linear rate equation will allow us to use the form of Eq. (10) to calculate the flux. Comparing Eq. (21) with Eq. (10) gives the mapping

$$K_i = \frac{k_d[E_i]q_i}{1 + q_i} \quad , \quad K_{-i} = \frac{k_d[E_i]}{1 + q_i}, \tag{22}$$

where now the rates depend on the diffusion-limited rate k_d, the enzyme concentration and the equilibrium constant of the reaction. Finally, substituting Eq. (22) into the expression for the flux,

Eq. (10), yields an expression for flux of a pathway composed of these “perfect enzymes”, where q is replaced by q^I for any reactions coupled to the conversion of the external metabolites:

$$J = \frac{1}{D}\left([S_0]\prod_{i=1}^{n} q_i^I - [S_n]\right), \tag{23}$$

where

$$D = \sum_{i=1}^{n} \frac{1}{K_i(E_i, q_i^I)} \prod_{m=i}^{n} q_m^I. \tag{24}$$

Optimizing the enzyme concentrations

Expression (23) for the pathway flux depends on the concentrations of the enzymes $[E_i]$, which are unknown. To evaluate the performance of our candidate pathways, we wish to compare the maximal possible flux that they can sustain for a given set of values of the external metabolite concentrations. To do so, we assume that the total concentration of enzyme available to each pathway is fixed, and that the cell is free to distribute the enzymes across the reactions so as to maximize the total flux. Altering the distribution of enzymes along the pathway (e.g. by regulating expression levels of the corresponding genes) will alter the steady state flux as well as the metabolite concentrations, and so this optimization must be performed subject to the constraints on the intermediate concentrations. The task of calculating the flux of a pathway thus becomes an optimization problem in which the flux must be maximized subject to the constraint of a fixed total enzyme concentration, $[E]_T$, and the constraint that all steady state metabolite concentrations must fall within some prescribed range. That is, we maximize J^*, where

$$J^* = \begin{cases} J([E_i]) & \text{for} \quad S_{\min} \le [S_i] \le S_{\max} \\ 0 & \text{otherwise} \end{cases}$$

subject to the constraint of fixed total enzyme concentration, $\sum_i [E_i] = [E]_T$. This optimization was performed using Powell’s method[27]. Note that the total amount of enzyme is assumed to be the same regardless of the pathway length, i.e. for longer pathways there will be on average less enzyme available per step.

Alternative flux calculation: reversible Michaelis-Menten kinetics

The flux calculation employed in the main text arguably represents the maximum flux a linear pathway can sustain – it assumes that all enzyme catalyzed reactions are limited only by the diffusion of the substrates (in addition to allowing the distribution of enzymes along each pathway to be optimized so as to maximize the flux). This assumption implies that all enzymes are in their unsaturated regime, where metabolite concentrations are much smaller than their corresponding Michaelis-Menten constants: $[S_i] \ll K_M$. This is often not the case in metabolism, where metabolites are commonly present at concentrations an order of magnitude higher than the corresponding enzymatic K_M values[3]. The perfect enzyme rate thus neglects any form of substrate saturation or

product inhibition, features of physical importance in real enzymatic systems.

For high metabolite concentrations we expect to see saturation of the enzymatic conversion rate. For example, it has been measured that in glucose-fed *E. coli.*, approximately 59% of measured metabolites have a concentration 10-fold higher than their K_M value, demonstrating a trend towards saturation of most enzyme sites[3] (though note that the trunk pathway of lower glycolysis was an exception in this study, where substrate concentrations were very similar to their K_M values). The flux calculation in the main text also neglects product inhibition, which may be of importance in real biological systems. Here we show that the key features of our results are not affected when we employ an alternative flux calculation that accounts for both saturating kinetics and product inhibition. In particular, we replace the assumption of linear reaction rates with perfect catalysis by a reversible Michaelis-Menten model.

We start by assuming the enzymatic mechanism

$$E + S \underset{k_{-1}}{\overset{k_1}{\rightleftharpoons}} ES \underset{k_{cat}^{-}}{\overset{k_{cat}^{+}}{\rightleftharpoons}} EP \underset{k_{-3}}{\overset{k_3}{\rightleftharpoons}} E + P \tag{25}$$

which includes the possibility of substrate saturation and product inhibition. From this mechanism an expression for the net reaction flux v, known as the reversible Michaelis-Menten equation, can be derived[28]:

$$v = \frac{d[P]}{dt} = \frac{V_f}{K_S} \left(\frac{1}{1 + \frac{[S]}{K_S} + \frac{[P]}{K_P}} \right) \left([S] - \frac{[P]}{q} \right) \tag{26}$$

where $q = [P]_{eq}/[S]_{eq} = e^{\frac{-\Delta G^0}{RT}}$ is the equilibrium constant of the reaction and $V_f = k_{cat}^{+}[E]_T$ is the maximal rate in the forward direction. In Eq. (26) K_S and K_P are the dissociation constants of the substrate and product respectively. Note that as in section 5, we choose not to model association and dissociation of external metabolites explicitly, but instead we incorporate their fixed concentrations into the equilibrium constant. In this way, each enzyme is characterized by 3 parameters: V_f, K_S and K_P.

In principle, one would like to allow for different parameter values for all enzymes in the various pathways, and also to optimize for enzyme concentrations, as we did for the calculations in the main text. However this is not feasible from a computational point of view, because we no longer have an analytic solution for the pathway flux and the pathway fluxes using Eq. (26) must be calculated numerically. To explore the relative performance of our pathways using the reversible Michaelis-Menten kinetic model, we therefore choose to assume that all enzymes have equal values of the parameters V_f, K_S and K_P. We also assume an equal distribution of enzyme concentration across all reactions in a pathway. We have previously found that this assumption has little effect on our results for the perfect enzyme. We investigate 2 specific scenarios.

Figure S3 shows the result of our comparison for the same glycolytic pathways shown in Figure 2 of the main text, when all enzymes are described by identical parameter values; $V_f = 10^{-3}$ M s^{-1}, $K_S = K_P = 10^{-5}$ M. Comparing to Fig. 2 of the main text, we find that the outcome is altered with respect to the ordering of pathways when averaged across the entire parameter space (left plot), but that the performance of the real trunk pathway (path 1, black) is still highest, both overall and in the physiological region of parameter space (right plot). The relative performance of

path 2 (green, path B in Fig. 4 main text) has improved, with it now performing best over a wider region of parameter space.

Setting $K_S = K_P$ above assumes that the enzyme has the same affinity for the substrate and product metabolites. Figure S4 repeats the above analysis for the case where $K_S = 10^{-6}$ M and $K_P = 10^{-3}$ M, i.e. the enzyme now has a higher affinity for the substrate metabolite than the product. This decreases the effect of product inhibition, and can be interpreted as each enzyme favouring the forward reaction. Again we see that although the rank order of the pathways is changed, the real pathway still performs best under typical physiological conditions, both when averaged across the entire parameter space (left plot) and in the physiological parameter region (right plot). Hence the key result of our study does not strongly depend on our choice of flux calculation: when realistic biophysical constraints are imposed on the intermediate metabolite concentrations, the real trunk pathway performs best under typical physiological conditions.

Sampling the parameter space

To evaluate the relative performance of our candidate pathways (Fig. 2 in the main text), we sampled the space of external metabolite concentrations, together with the concentrations of G3P and pyruvate in the following way. The parameter set was defined as in Table S4; the ratios [ATP]/[ADP] and [NAD]/[NADH], for example, are treated as single parameters since they occur in these combinations in the flux expression. Each parameter was sampled logarithmically over a wide range, covering several orders of magnitude above and below its typical physiological concentration; the ranges sampled are given in Table S4. The results of our work are robust to the choice of parameter range. Indeed, if we restrict these ranges to a tighter region around the physiological values (i.e. red rectangle) we find that the real pathways are ranked higher relative to the alternatives, as is clear from Fig. 2 in the main text.

---

[1] Ronimus, R. S. & Morgan, H. W. Distribution and phylogenies of enzymes of the embden-meyerhof-parnas pathway from archaea and hyperthermophilic bacteria support a gluconeogenic origin of metabolism. *Archaea* **1**, 199–221 (2002).

[2] Berg, J., Tymoczko, J. & Stryer, L. *Biochemistry* (W H Freeman and Company, 2002), 5th edn.

[3] Bennett, B. D. *et al.* Absolute metabolite concentrations and implied enzyme active site occupancy in escherichia coli. *Nat. Chem. Biol.* **5**, 593–599 (2009).

[4] Noor, E. *et al.* Pathway thermodynamics highlights kinetic obstacles in central metabolism. *PLoS Computational Biology* **10** (2014).

[5] Flamholz, A., Noor, E., Bar-Even, A., Liebermeister, W. & Milo, R. Glycolytic strategy as a tradeoff between energy yield and protein cost. *Proc. Natl Acad. Sci. USA* **110**, 10039–10044 (2013).

[6] Phillips, S. A. & Thornalley, P. J. The formation of methylglyoxal from triose phosphates. *European Journal of Biochemistry* **212**, 101–105 (1993).

[7] Haraldsdôttir, H., Thiele, I. & Fleming, R. Quantitative assignment of reaction directionality in a multicompartmental human metabolic reconstruction. *Biophysical Journal* **102**, 1703–1711 (2012).

[8] Bar-Even, A., Flamholz, A., Noor, E. & Milo, R. Rethinking glycolysis: on the biochemical logic of metabolic pathways. *Nat. Chem. Biol.* **8**, 509–517 (2012).

[9] Bustos, D. M., Bustamante, C. A. & Iglesias, A. A. Involvement of non-phosphorylating glyceraldehyde-3-phosphate dehydrogenase in response to oxidative stress. *Journal of Plant Physiology* **165**, 456–461 (2008).

[10] Verhees, C. H. *et al.* The unique features of glycolytic pathways in archaea. *Biochem. J.* **375**, 231–246 (2003).
[11] Ahmed, H. *et al.* The semi-phosphorylative Entner-Doudoroff pathway in hyperthermophilic archaea: a re-evaluation. *Biochem. J.* **390**, 529–540 (2005).
[12] Kouril, T. *et al.* Intermediate instability at high temperature leads to low pathway efficiency for an in vitro reconstituted system of gluconeogenesis in *Sulfolobus solfataricus*. *FEBS Journal* **280**, 4666–4680 (2013).
[13] Zhoua, J. *et al.* Atypical glycolysis in *Clostridium thermocellum*. *Applied and Environmental Microbiology* **79**, 3000–3008 (2013).
[14] Thornalley, P. J., Langborg, A. & Minhas, H. S. Formation of glyoxal, methylglyoxal and 3-deoxyglucosone in the glycation of proteins by glucose. *Biochem. J.* **344**, 109–116 (1999).
[15] Webb, E. *Enzyme nomenclature 1992: recommendations of the Nomenclature Committee of the International Union of Biochemistry and Molecular Biology on the nomenclature and classification of enzymes* (Academic Press, San Diego, 1992), 6 edn.
[16] Mavrovouniotis, M. L. Estimation of standard gibbs energy changes of biotransformations. *Journal of Biological Chemistry* **266**, 14440–14445 (1991).
[17] Mavrovouniotis, M. L. Group contributions for estimating standard gibbs energies of formation of biochemical compounds in aqueous solution. *Biotechnology and Bioengineering* **36**, 1070–1082 (1990).
[18] Jankowski, M. D., Henry, C. S., Broadbelt, L. J. & Hatzimanikatis, V. Group contribution method for thermodynamic analysis of complex metabolic networks. *Biophys. J.* **95**, 1487–1499 (2008).
[19] Noor, E. *et al.* An integrated open framework for thermodynamics of reactions that combines accuracy and coverage. *Bioinformatics* **28**, 2037–2044 (2012).
[20] Alberty, R. A. *Biochemical Thermodynamics: Applications of Mathematica*. Methods of Biochemical Analysis (2006).
[21] Alberty, R. A. Calculation of standard transformed entropies of formation of biochemical reactants and group contributions at specified pH. *The Journal of Physical Chemistry A* **102**, 8460–8466 (1998).
[22] Alberty, R. Chemical thermodynamic properties of isomer groups. *Industrial & Engineering Chemistry Fundamentals* **22**, 318–321 (1983).
[23] Heinrich, R., Montero, F., Klipp, E., Waddell, T. G. & Melẽndez-Hevia, E. Theoretical approaches to the evolutionary optimization of glycolysis: Thermodynamic and kinetic constraints. *Eur. J. Biochem.* **243**, 191–201 (1997).
[24] Heinrich, R. & Hoffmann, E. Kinetic parameters of enzymatic reactions in states of maximal activity; an evolutionary approach. *J. theor. Biol* **151**, 249–283 (1991).
[25] Albery, J. W. & Knowles, J. R. Evolution of enzyme function and the development of catalytic efficiency. *Biochemistry* **15**, 5631–5640 (1976).
[26] Pettersson, G. Effect of evolution on the kinetic properties of enzymes. *Eur. J. Biochem.* **184**, 561–566 (1989).
[27] Press, W. H., Teukolsky, S. A., Vetterling, W. T. & Flannery, B. P. *Numerical Recipes: The Art of Scientific Computing* (Cambridge University Press, New York, NY, USA, 2007), 3rd edn.
[28] Noor, E., Flamholz, A., Liebermeister, W., Bar-Even, A. & Milo, R. A note on the kinetics of enzyme action: A decomposition that highlights thermodynamic effects. *FEBS Letters* **587**, 2772–2777 (2013). A century of Michaelis-Menten kinetics.
